# Evolutionary Adaptations of Parasitic Flatworms to Different Oxygen Tensions

**DOI:** 10.3390/antiox11061102

**Published:** 2022-05-31

**Authors:** José de Jesús Martínez-González, Alberto Guevara-Flores, Irene Patricia del Arenal Mena

**Affiliations:** Departamento de Bioquímica, Facultad de Medicina, Universidad Nacional Autónoma de México, Ciudad de Mexico 04510, Mexico; jjmtz@bq.unam.mx (J.d.J.M.-G.); guevarafa@bq.unam.mx (A.G.-F.)

**Keywords:** platyhelminthes, Cestoda, oxygen tension, anaerobic metabolism, tegument, mitochondria

## Abstract

During the evolution of the Earth, the increase in the atmospheric concentration of oxygen gave rise to the development of organisms with aerobic metabolism, which utilized this molecule as the ultimate electron acceptor, whereas other organisms maintained an anaerobic metabolism. Platyhelminthes exhibit both aerobic and anaerobic metabolism depending on the availability of oxygen in their environment and/or due to differential oxygen tensions during certain stages of their life cycle. As these organisms do not have a circulatory system, gas exchange occurs by the passive diffusion through their body wall. Consequently, the flatworms developed several adaptations related to the oxygen gradient that is established between the aerobic tegument and the cellular parenchyma that is mostly anaerobic. Because of the aerobic metabolism, hydrogen peroxide (H_2_O_2_) is produced in abundance. Catalase usually scavenges H_2_O_2_ in mammals; however, this enzyme is absent in parasitic platyhelminths. Thus, the architecture of the antioxidant systems is different, depending primarily on the superoxide dismutase, glutathione peroxidase, and peroxiredoxin enzymes represented mainly in the tegument. Here, we discuss the adaptations that parasitic flatworms have developed to be able to transit from the different metabolic conditions to those they are exposed to during their life cycle.

## 1. Introduction

A crucial moment in the evolution of the planet was the change from an anoxic primordial atmosphere to one rich in oxygen (O_2_). Currently, it is accepted that this change began with the origin of cyanobacteria and the development of oxygenic photosynthesis [1,2] and continued later with the emergence and diversification of photosynthetic pigments in different types of algae and plants [3,4]. The latter allowed the atmosphere to accumulate oxygen over millions of years and, after some fluctuations in the Carboniferous period, to reach its current level (around 21%) [5,6]. In turn, this process influenced the evolution of life on Earth, since geological and fossil evidence has allowed us to infer that the increase and accumulation of O_2_ in the atmosphere gave rise to the establishment of specific ecological niches. Hence, some of the organisms adapted and developed in the increasingly aerobic conditions, whereas others established themselves in a microaerophilic environment and still others in sites where fully anaerobic conditions predominated [7,8]. There is even a proposal that considers that the first organisms to emerge were anaerobes, which allowed them to adapt and eventually live in hypoxic conditions [9,10]. In fact, it is recognized that anaerobic glycolysis is an ancestral metabolic pathway as it is present in these first living beings, which in turn allowed them to produce ATP at substrate-level phosphorylation in the absence of O_2_ [11,12]. Moreover, the late oxygen accumulation in the atmosphere caused the emergence of aerobic-type energy metabolism, in which the now available O_2_ is the final electron acceptor, enabling the ability to obtain a greater amount of ATP from a glucose molecule. This would favor the appearance and diversification of new metabolic pathways [13], which in turn enabled organisms to evolve into more complex forms.

Oxygen is one of the two main products derived from oxygenic photosynthesis. This molecule is chemically interesting since it has two unpaired electrons in the anti-union orbitals (with the same spin), which makes it difficult for it to oxidize another molecule and accept two electrons simultaneously [14]. This restriction in molecular oxygen (that is, the dioxygen di-radical) significantly decreases its reactivity; however, exposure to physical factors such as high temperatures or some source of radiation can cause a change in the spin, thereby decreasing said restriction, which favors the acceptance of one electron at a time (that is, oxygen becomes more reactive), making reactions very slow [15]. Due to this, an incomplete reduction of O_2_ can occur, generating a series of molecules that, when accumulated, can cause adverse effects in the cell. These molecules are generically called reactive oxygen species (ROS); they include the superoxide radical anion (O_2_^•−^), hydrogen peroxide (H_2_O_2_), and the hydroxyl radical (HO^•^), among others.

The purpose of this review is to analyze some of the basic adaptations presented by parasitic flatworms, a specific group of organisms that face changes in the concentration of oxygen (and related molecules) throughout their life cycle.

## 2. Parasitic Flatworms and Oxygen Availability

### 2.1. General Information about Flatworms

The Platyhelminthes, also known as flatworms, are dorsoventrally flattened organisms with bilateral symmetry [16] that morphologically constitute a heterogeneous group. Throughout history, the phylum Platyhelminthes has been the subject of multiple controversies, especially regarding its phylogeny since morphological data of some species can be troublesome [17]. We currently have nuclear and mitochondrial genomes and transcriptomic analyses that allow us to reach a consensus. The phylum Platyhelminthes is currently included within the Lophotrochozoa supergroup, which includes other invertebrates, as well as annelids and mollusks [18,19]. It is interesting to note that another phylum, which was traditionally thought to be close to flatworms, the phylum Nematoda, is included, together with arthropods and related groups, in the supergroup Ecdysozoa [18]. This is highly important to consider to understand the morphophysiological differences between flatworms and nematodes.

Already within the phylum Platyhelminthes is the paraphyletic group Turbellaria, which includes all free-living flatworms. These are found in aqueous environments, both in salt and fresh water, although there are some adapted to terrestrial environments with high humidity. An important characteristic that is present in the members of this group is that their external surface has a simple epidermis, composed of a single layer of columnar epithelium located on top of a basement membrane and several layers of muscle. This epithelium usually has cilia, which are used by these worms to swim in waterbodies or to glide over the substrate [20].

On the other hand, we have the monophyletic group Neodermata, whose innovation is replacement of the simple epidermis of the turbellarians by the presence of a syncytial-type tegument that is formed by extensions of cells that are below the basement membrane and that fuse together in the tegument creating a syncytium. This characteristic is considered an adaptation to parasitic life and is so important to understand their physiology that it is a criterion for defining this group, which is composed exclusively of parasitic organisms. Three types of flatworms with a clearer phylogenetic relationship are currently recognized [21]. On the one hand, we have the group of ectoparasitic flatworms of the class Monogenea, whose representatives are characterized by having a single host throughout their life cycle [22]; they usually live on the gills or skin of aquatic vertebrate animals. On the other hand, we have the flatworms that are endoparasites and are grouped within the class Cestoda and class Trematoda. These last two are considered the most successful parasites due to the great variety of vertebrates they infect; they are responsible for many diseases of livestock animals and humans [23,24,25]; thus, they are of great medical and economic relevance [26,27].

### 2.2. The Complex Life Cycles of the Trematoda and Cestoda

This group of organisms presents complex biological cycles in which some may have free-living stages or need one or more intermediate hosts of invertebrate or vertebrate origin to finally invade a definitive host in which they move until they find the tissue or organ, where they settle and reproduce sexually.

Trematodes, also known as flukes, have as their main characteristic the retention of the cecum, although they can also absorb nutrients and carry out gas exchange through their body wall (also called tegument) [28]. Their first intermediate hosts are generally mollusks, and adults can have a wide variety of diets, from blood to epithelia [29]. Some of the species in which pioneering studies on the biochemistry and immunology of trematodes have been made belong to this group: *Clonorchis sinensis*, *Fasciola hepatica*, and *Schistosoma mansoni*.

Cestodes are one of the most successful groups within the parasitic flatworms. This is due in part to their complete adaptation to parasitic life, including the total absence of an internal digestive system, the lack of an intermediate free-living form, the appearance of structures specific for attachment to the intestine of the definitive host, and serial repetition of a hermaphroditic reproductive complex [30]. This group has been problematic to study due to the difficulty in accessing and maintaining the biological material, the fragility of the specimens outside their hosts, and the contradictory information from the first studies [17]. Within this group, we have well-known species such as *Hymenolepis diminuta*, *Echinococcus granulosus*, and *Taenia solium*.

**Figure 1:**
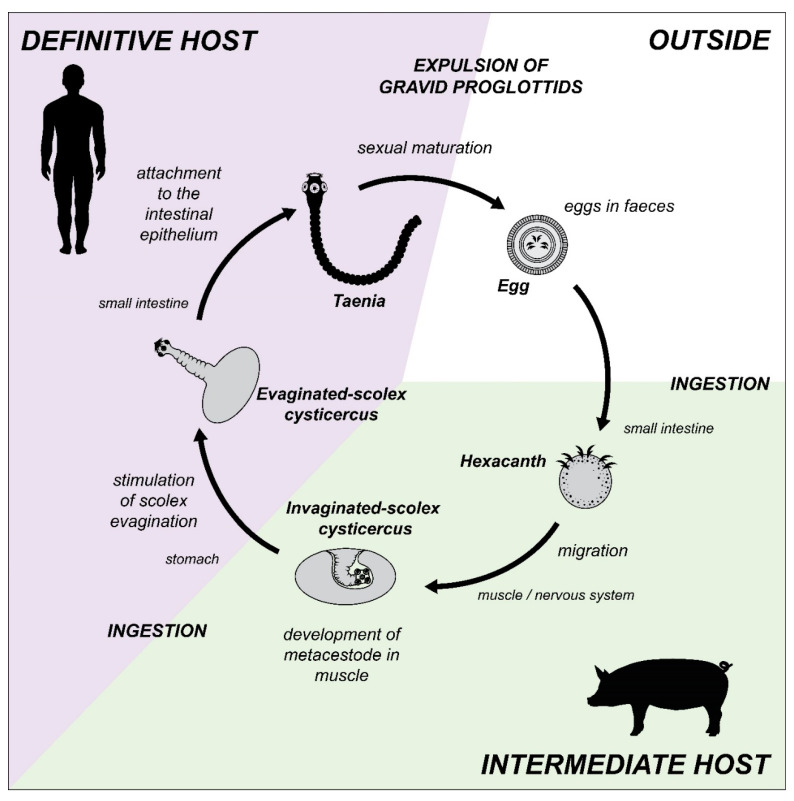
Life cycle of *Taenia solium.* Parasite stages and its migration through the interior of its intermediate host (e.g., pig, in green) and its definitive host (e.g., man, in purple), is illustrated.

A representative life cycle of this group is found in *T. solium* (Figure 1). After the ingestion of feces contaminated with embryonated eggs (also called hexacanth larva) by a pig, the protective cover of such eggs is eliminated and the larval oncosphere form emerges. This oncosphere crosses the intestinal mucosa and migrates through the systemic circulation to lodge in various tissues, with a preference for the skeletal muscle and the nervous system. Already there, the larva develops to its metacestode form (also known as cysticercus), where it can stay for years, asymptomatically. Finally, when the definitive host (man) ingests pork meat contaminated with cysticerci, it carries out its last metamorphosis, which is a distinctive characteristic of cestodes [31]. It consists in the activation of the larva by means of pepsin and stomach acid, as well as the bile salts, of the definitive host, causing evagination of a fixing structure, the scolex, which will allow it to anchor itself to the intestinal epithelium. At this point, the adult tapeworm form rapidly begins to develop and matures sexually to generate a series of hermaphrodite structures called proglottids, each of which contains a complete set of male and female reproductive organs that mate with their other proglottid counterparts. Eventually they are filled with millions of fertile eggs (becoming gravid proglottids) that will detach and leave the host along with the feces to later be eaten by the pig to close the life cycle. Occasionally, man can accidentally ingest the eggs of *T. solium* that give rise to the development of the larva (metacestode) and that produces cysticercosis.

**Figure 2:**
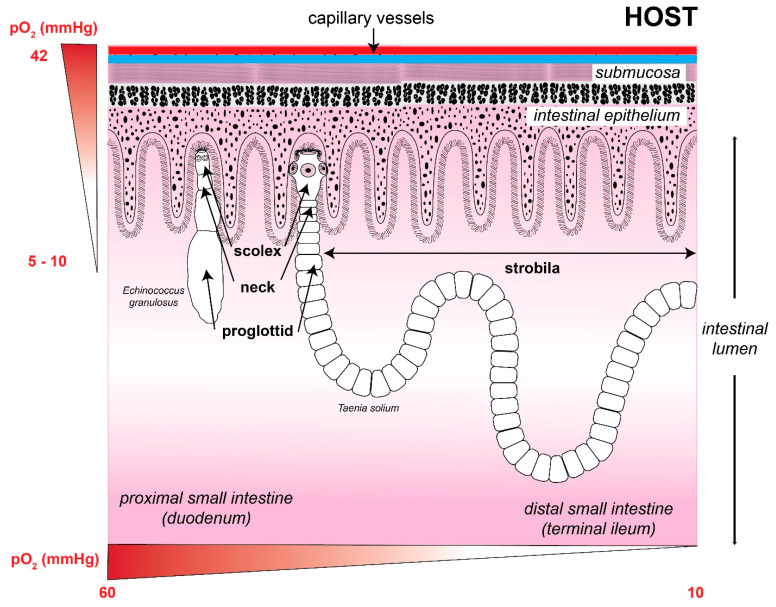
Adult form of the cestodes is exposed to the intestinal oxygen concentration. The image shows a structural drawing of the adult tapeworm form of *Echinococcus granulosus* (left; size range 2–7 mm) and *Taenia solium* (right; size range 2–7 m), attacking the intestinal epithelium of their definitive host. Oxygen tension in the intestinal tissue decreases the further away parasites are from the intestinal capillaries, while the oxygen concentration in the intestinal lumen decreases as parasites move towards the colon, where the environment is practically anaerobic. Oxygen concentrations were obtained from references [33,34,35,36]. The size of the parasites was obtained from the Laboratory Identification of Parasites of Public Health Concern website (https://www.cdc.gov/dpdx/ (accessed on 17 February 2022)).

At this point, it is possible to assume that, during their free-living phase (in the case of trematodes) or the intermediate step between hosts, these endoparasitic flatworms are exposed to a higher O_2_ tension that they can face inside the hosts cells, hence, their energy metabolism will preferably be aerobic [32]. During their transit and accommodation in the host, they are exposed to variable concentrations of O_2_, so their anaerobic energy metabolism is expected [33,34,35,36] (Figure 2 and Table 1). For example, in their adult state, tapeworms settle in the intestine of their vertebrate host. In this organ, the partial pressure of O_2_ (pO_2_) can vary from 0 to 16 mmHg, with a three-times higher pressure in the mucosa than in the intestinal lumen, where it can reach zero [37]. Additionally, the presence of O_2_ is also modified according to the postprandial state because an increase has been observed in O_2_ when the digestion process begins as well as in this tissue’s blood supply [38].

The aforementioned indicates that the different habitats occupied by these parasites during their life cycle determine their energy metabolism and their transition from an aerobic to an anaerobic metabolism [39,40,41]. An example of this adaptation has been reported in trematodes such as *F. hepatica*. It was observed that its free-living larva has an aerobic metabolism, but when it invades the bile ducts of the vertebrate host, its metabolism is basically anaerobic [42,43] (see below).

## 3. Adaptations of Parasitic Flatworms to Changes in Oxygen Tension

### 3.1. Ultrastructural Adaptations

#### 3.1.1. Body Wall (Tegument)

**Figure 3:**
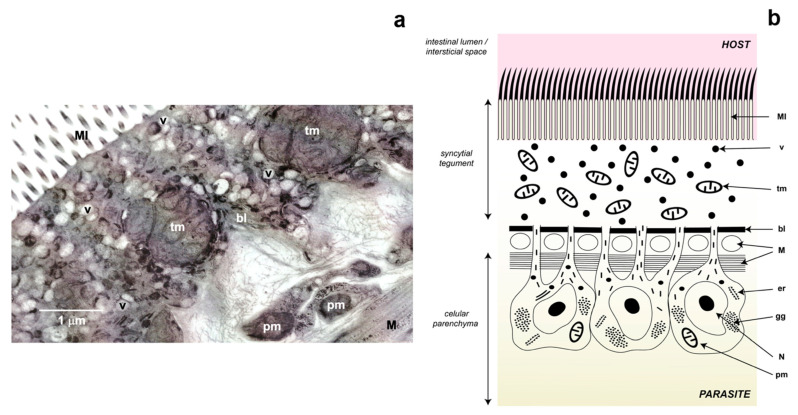
Tegument of the cestodes. Panel (**a**) represents a photograph of the tegument of *Taenia crassiceps* cysticercus obtained by transmission electron microscopy. Panel (**b**) is a schematic representation of the tegument of cestodes. *Abbreviations*: bl, basal lamina; er, endoplasmatic reticulum; gg, glycogen granules; M, muscle; MI, microtriches; N, nucleus; pm, parenchymal mitochondria (anaerobic mitochondria); tm, tegumental mitochondria (aerobic mitochondria); v, vesicles.

The parasitic flatworms of the Neodermata group have a glycocalyx rich in carbohydrates in their external part of the membrane that limits the tegument, which consists of a simple syncytium that covers the entire surface of these worms [44]. However, this tissue results from the fusion of cytoplasmic projections of parenchymal cells (also known as cytones) that are found below the basement membrane and whose function is to provide a constant flow of proteins and other molecules to the tegument [45] (Figure 3). Ultrastructural adaptations can be present such as microtriches in the case of cestodes, which increase the surface area of the parasite allowing a greater exchange between it and the host, as well as recognition mechanisms through the glycocalyx [46].

In the case of the phylum Platyhelminthes, due to their flattened morphology, gas exchange as well as nutrient uptake can take place through the body wall because these organisms lack a circulatory system. Naturally, the uptake of O_2_ occurs by simple diffusion and is carried out through this structure. At this point, it is important to note that there is a gradient in the concentration of oxygen in the parasite, where the tegument, being the most exposed region, presents the highest pO_2_, whereas the oxygen concentration decreases when entering the internal tissues of the parasite [47]. In addition to this, there is an important relationship with the size of the parasite, as in *F. hepatica* [48].

The tegument is essential for the success of these parasites and, in fact, it plays a key role in the evolution of parasitism in these animals due to the inseparable host-parasite bond that is generated [49]. In practice, it is a barrier that protects the parasite from the host’s immune system [50,51] and from the hostile conditions in the digestive tract, blood, or other organs [46]. Additionally, it serves to house the molecular systems that will serve multiple purposes such as migration through the host’s body, antioxidant defense, repair of damage caused by the attack of the immune system, and evasion and modulation of the immune system response [52]. We will deal with these points at the end of this review.

#### 3.1.2. Diversity of Mitochondria

Parallel to the appearance and enrichment of O_2_ in the atmosphere and, consequently, the diversification of living organisms, diverse types of mitochondria were also generated, from mitoplasts to aerobic mitochondria [53,54].

Palade in 1953 [55] recognized that variation in the size, shape, and internal organization of mitochondria seems to reflect their physiological and biochemical differences in different cells of an organism. It is now well known that tissues with a high demand in their energy metabolism contain several hundred mitochondria per cell and that they have many densely packed cristae, whereas in tissues with a lower energy demand, mitochondria with fewer cristae and smaller in size are present [56].

**Figure 4:**
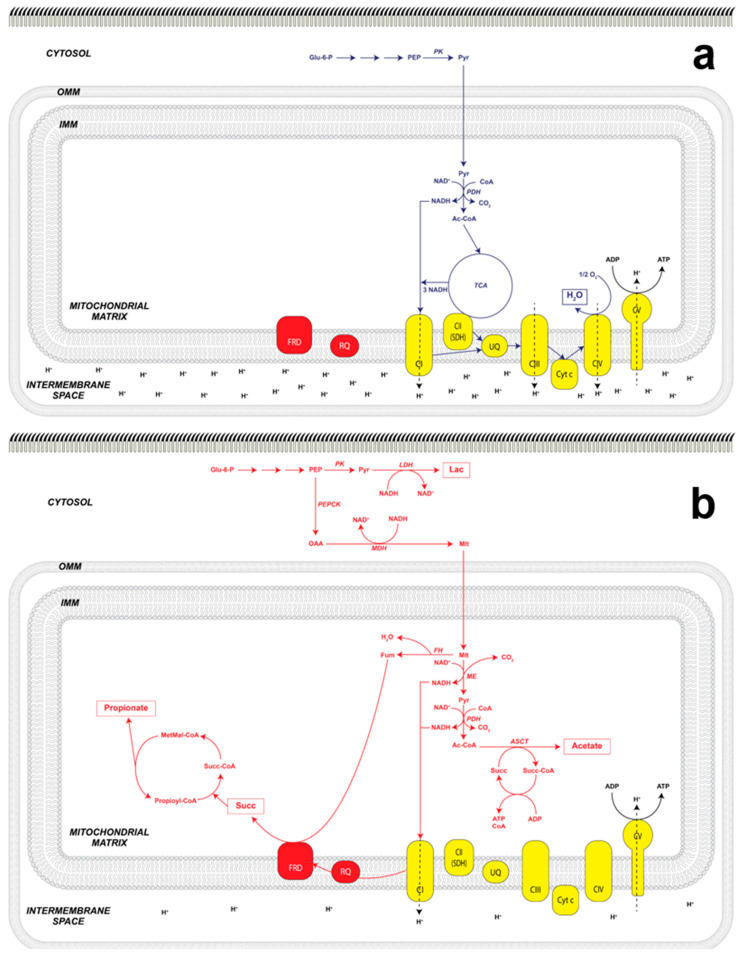
Aerobic and anaerobic energy metabolism in parasitic flatworms. Representation of the electron flow in aerobic metabolism: Panel (**a**), while Panel (**b**), represents the electron flow corresponding to anaerobic metabolism. Abbreviations: OMM, outer mitochondria membrane; IMM, inner mitochondria membrane; Ac-CoA, Acetyl coenzyme A; ASCT, acetate succinate-CoA transferase; cyt*c*, cytochrome *c*; Fum, fumarate; FH, fumarate hydratase (fumarase); FRD, fumarate reductase; Glu-6-P, glucose 6-phosphate; Lac, lactate; LDH, lactate dehydrogenase; MDH, malate dehydrogenase; ME, mitochondrial malic enzyme; MetMal-CoA, methylmalonyl coenzyme A; Mlt, malate; PEP, phosphoenol pyruvate; PEPCK, phosphoenol pyruvate carboxykinase; PDH, pyruvate dehydrogenase complex; PK, pyruvate kinase; Pyr, pyruvate; OAA, oxaloacetate; RQ, rhodoquinone; Succ, succinate; Succ-CoA, succinyl-coenzime A; SDH, succinate dehydrogenase; TCA, tricarboxylic acid cycle; UQ, ubiquinone.

According to their metabolism, two large groups of mitochondria can be distinguished: aerobic and anaerobic. Aerobic mitochondria in the presence of O_2_ carry out the Krebs cycle and oxidative phosphorylation. In contrast, anaerobic mitochondria are structurally similar to typical mitochondria but function in the absence of O_2_; although their enzymatic repertoire is not very different from that of aerobic mitochondria [9,57], because many of these enzymes can catalyze the reverse reaction under certain conditions. Some enzymes of the Krebs cycle participate in these two metabolic pathways, such as fumarase and succinyl-CoA synthetase, whereas other enzymes participate in anaplerotic pathways such as phosphoenol-pyruvate kinase (PEPK) and mitochondrial malic enzyme (mME) (Figure 4). The above allows these two metabolisms, aerobic and anaerobic, to occur almost simultaneously or in different regions of a parasite, with pO_2_ ultimately determining their prevalence [58].

Regarding the pO_2_ to which they are exposed, structurally and metabolically different mitochondria have been described in the same organism related to the gradient that is established when O_2_ diffuses from the tegument towards the cell parenchyma [59]; thus, it is expected that the tegument presents a higher concentration of O_2_ than the parenchyma [60]. In 1967, Lumsden [61] described the presence of a heterogeneous population of mitochondria in the cestode *Lacistorhynchus tenuis*, where he reports that the parenchyma cells are larger despite occurring in smaller numbers and having fewer cristae compared to mitochondria of the tegument. This differential distribution of the types of mitochondria in the tissues of the cestodes was subsequently corroborated in the *Taenia crassiceps* metacestode [62], where we determined the aerobic metabolism of the mitochondria present in the tegument of the cysticercus and were able to observe that in addition to being very numerous, they have highly developed cristae (Figure 3). The presence of aerobic mitochondria in the tegument is not exclusive to cestodes; Takamiya observed the presence of several types of mitochondria in the trematode *Paragonimus westermani* [63]. On the one hand, in the tegument, he described numerous mitochondria with a larger number of cristae and a greater amount of cytochrome *c* oxidase activity (a marker of aerobic metabolism) compared to that of the parenchyma cells, where this author reported the presence of two types of mitochondria similar in size but one being completely anaerobic and the other one only partially so.

### 3.2. Metabolic Adaptations

#### 3.2.1. Oxygen Carriers and Storage

Due to its active metabolism and energy needs that require a high production of eggs, a determining factor in the biology of parasitic flatworms is the availability of oxygen, both in trematodes and in cestodes.

As a consequence of the low solubility of O_2_ in aqueous medium [64], some animals have developed a whole repertoire of respiratory pigments, which are metalloproteins and whose metallic element allows them to bind to O_2_ temporarily to transport it through the circulatory system and to distribute it in body tissues [65]. Thus, for example, mammals depend on myoglobin (Mb), a monomeric protein associated mainly with cardiac and skeletal muscle, and on hemoglobin (Hb), a tetrameric protein contained in erythrocytes; both present a coordinated iron atom in a tetrapyrrolic chemical structure [66].

Several homologues of myoglobins have been reported in vertebrates: androglobin (Adgb), neuroglobin (Ngb), globin X (GbX), myoglobin (Mb), and cytoglobin (Cygb) [67]. However, these present an unequal distribution among the groups of flatworms. In the case of the trematodes, *F. hepatica* and *P. westermani*, an Mb with a high affinity for O_2_ and a low *K*_d_, was initially described [68], similar to that reported in the nematode *Ascaris suum* [69,70]. In the case of cestodes, until 2002, Mb could be co-purified in the cysticercus of *T. solium*, possibly associated with muscle fibers of the subtegumental area [71]. Currently, through genomic and phylogenetic analyses, the presence of globins belonging to each of the GbX, Ngb, and Mb subfamilies was deduced in trematodes, whereas the cestode species had an Ngb-like single protein, and in turbellarians it was not possible to detect this subfamily, but the GbX and Mb-like proteins [72] were identified.

It is widely accepted that myoglobins, due to their high affinity for O_2_, function mainly as storage rather than for oxygen transport [73]. However, other additional functions have begun to be proposed for this type of molecule, such as reserves of the heme group (associated with egg production) or to serve as a ROS detoxifier [72]. For example, in the adult trematode *C. sinensis*, five different types of globins (CsMb 1–5) were identified, of which only CsMB-1 was found in the subtegumental area (parenchyma) and was the only one to respond to stimuli by exogenous O_2_, whereas the other globins were located exclusively in sexual organs and intrauterine eggs, which supports the participation of some globins in other specialized non-respiratory tasks.

Nevertheless, a high pO_2_ at the parasite’s site does not necessarily imply that aerobic metabolism is present. An example of this is observed in *S. mansoni*, whose adult form lodges in the portal vein and whose metabolism has been reported to be preferably anaerobic, although this is not exclusive [74,75]. In contrast, the exceptionally high affinity for oxygen presented by the Hb of the fluke *Ophisthorchis viverrini* allows it to live in a practically anaerobic environment, such as the bile ducts [76].

#### 3.2.2. HIF and the Detection of Oxygen in the Environment

As mentioned above, in most mammalian tissues there is between 2% and 9% O_2_ (an average of 40 mm Hg). In this sense, hypoxia is usually defined as ≤2% O_2_, whereas severe hypoxia (or anoxia) is defined as ≤0.02% O_2_ [77]. Therefore, it is important to have mechanisms that detect pO_2_ in real time to allow the cell to respond appropriately. The change in oxygenation is sensed by HIF (hypoxia-inducible factor), a transfer factor that in hypoxic conditions induces the change from aerobiosis to anaerobiosis. This, in turn, brings about a cascade of events such as the overexpression and activation of enzymes like lactate dehydrogenase (LDH), which is necessary for lactate formation and, simultaneously, activating pyruvate dehydrogenase kinase (PDHK) to prevent pyruvate production [58].

Apparently, the mechanism mediated by HIF and related proteins is highly conserved in animals, making it possible to detect cnidarians and sponges even in basal groups of metazoans [78]. In its active form, HIF is a heterodimer consisting of HIF-1α and HIF-1β. In the case of parasitic flatworms, it is expected that, due to their exposure to different concentrations of O_2_, during their life cycles, HIF plays a relevant role. In fact, the HIF-1α and HIF-1β proteins have already been characterized in the parasitic nematode *A. suum* [79]. However, it was not until 2019 that a HIF-1α homologue (as well as other associated genes) could be isolated and characterized in the trematode *C. sinensis* (CsHIF-1α) [80]. As expected, CsHIF-1α was highly induced in adults under hypoxic conditions in vitro. Interestingly, CsHIF-1α was sensitive to changes in nitrite and nitric oxide, hence, the authors suggest that these molecules, together with O_2_, participate in the induction of the response to hypoxia in this organism.

#### 3.2.3. Aerobic Metabolism

In principle, we must recognize that glucose is the main source of energy used by parasitic flatworms (both in adult and larval forms [81]), while glycogen formation is their main energy conservation strategy [82,83,84,85,86,87]. In fact, in the case of glycogen, it has been previously identified through histochemical techniques and later through transmission electron microscopy, where many glycogen granules are observed that can serve in cestodes as a source of energy in fasting situations as reported in *T. solium* tapeworms [88] and the metacestode (traditionally also known as *Cysticercus cellulosae*) [89].

Therefore, under aerobic conditions, these organisms will use the traditional pathways to obtain reducing power through the oxidation of glucose (glycolysis and the Krebs cycle) and the subsequent production of energy coupled to oxidative phosphorylation (OXPHOS) (Figure 4) [90]. In both trematodes [91] and cestodes [92], it has been possible to identify the genes encoding enzymes of each of these pathways at the genome level. However, transcriptomic analyses have shown a differential expression of these enzymes in the tissues of the parasite, as demonstrated in the metacestode of *Echinococcus granulosus* in which the expression of enzymes from fermentative pathways associated with the germ layer and the gluconeogenic pathway associated with both the germinal layer and the protoscolex were detected [83]. Additionally, the expression can be influenced by experimental conditions. Fraga et al. [93] were able to successfully detect all the metabolites associated with the Krebs cycle in the metacestode of *T. crassiceps* under in vivo conditions; thus, it was inferred that this pathway is complete in the cysticercus. In this same parasite, we characterized the aerobic metabolism in the mitochondria of the tegument [62,94].

In the case of the metabolic pathways of lipids and proteins, there are great changes, which may be due to the adaptation to the conditions of a parasite related to what the host provides. In 2013, Tsai and a large team of collaborators reported the massive sequencing and comparison of four cestode genomes (*E. granulosus*, *Echinococcus multilocularis*, *Hymenolepis microstoma*, and *T. solium*) [92]. Basically, they reported a significant reduction in the metabolic capacity of these organisms, as well as the presence of specialized elements in the uptake of nutrients.

#### 3.2.4. Anaerobic Metabolism

Glycolysis can be considered a universal pathway by which many organisms can obtain energy. Its final product, pyruvate (Pyr), can be used in other alternative pathways known as fermentative pathways; they occur in the absence of O_2_ and allow the NADH generated during glycolysis to be oxidized to NAD^+^, a necessary substrate for, so that this path can continue (Figure 4).

A classic adaptation of anaerobic metabolism is lactate fermentation, in which pyruvate is reduced to lactate (Lac) by the enzyme lactate dehydrogenase (LDH) using electrons from NADH. It is now known that this pathway is also used in parasitic helminths. Direct evidence of its presence is the secretion of Lac into the medium, as has been reported in cestodes such as *Moniezia expansa* [95,96], *E. granulosus* [83], and *E. multilocularis* [86].

In addition to Lac secretion, the secretion of other reduced compounds such as succinate (Succ), acetate, and propionate (PPO) has been reported. This has been reported in *M. expansa* [32], and confirmed in *E. granulosus* [83], *T. crassiceps* [84], and *E. multilocularis* [86], where the main secreted product was succinate; these products are the result of a pathway known as malate dismutation (Figure 4).

Malate dismutation is the main anaerobic pathway present in parasitic platyhelminths [43,97,98,99], and has as its final products a reduced molecule and an oxidized (as occurs in the reactions called dismutation). During this process, phosphoenolpyruvate (PEP) produced during glycolysis is carboxylated to oxaloacetate (OAA) by PEP carboxykinase (PEPCK), producing ATP by substrate-level phosphorylation. OAA is reduced to malate (Mlt) through the cytosolic malate dehydrogenase (cMDH), which has NADH produced during glycolysis as another substrate. Subsequently, Mlt enters the mitochondria and, on the one pathway, through fumarase (also named fumarate hydratase, FH), it produces fumarate (Fum) and, by another, the mitochondrial malic enzyme (mME) oxidatively decarboxylates it to Pyr that can later generate acetate (Figure 4).

The fumarate produced is a substrate for the enzyme fumarate reductase (FDR) that reduces it to Succ; this is the main product of electron secretion in cestodes [95,96,100,101]. To not accumulate this metabolite and to maintain its redox balance, Succ is secreted into the surrounding medium, as reported in the culture medium of *T. crassiceps*, as well as in the cysticerci of *T. solium* removed from pig brains [84,96]. Additionally, it has been reported that Succ can generate PPO. Recently, two alternative pathways for propionate formation have been reported: (a) from succinyl-CoA to methylmalonyl-CoA that is decarboxylated to generate ATP and propionyl-CoA, which, in the presence of Succ, releases PPO and acetylates succinate; this process appears to occur under prolonged anaerobic conditions [102]; and (b) via Lac accumulation and its transformation to propionyl-CoA releasing PPO and regenerating CoA [103]. This contrasts with what Ritler et al. reported, after they could not detect propionate as a secretion product in *E. multilocularis* [86].

The other malate dismutation reaction is the one that produces acetate where, as mentioned, the pyruvate generated in the mitochondrial matrix, through the mME (and in addition NAD(P)H is generated) [98,101] and through the pyruvate dehydrogenase (PDH) complex, is oxidatively decarboxylated to generate acetyl-CoA and NADH. Finally, through the enzyme acetate-succinate-CoA transferase (ASCT), CoA is transferred to Succ, producing acetate and succinyl-CoA [104]. A search of available genomic databases indicates the presence of ASCT genes in the flukes *S. mansoni*, *P. westermani*, *C. sinensis*, and *F. hepatica* [105], as well as in the nematodes *Ostertagia ostertagi*, *Anisakis simplex*, and *Brugia malayi*. Although the presence of the gene encoding for ASCT in cestodes has not been reported, it is possible to suggest the presence of the enzyme (or an analogous pathway) since the presence of acetate has been reported as an end-product of anaerobic respiration in both *T. solium* [84] and *E. multilocularis* [86].

The NADH generated in the previous reactions transfers its electrons to the mitochondrial complex I NADH-rhodoquinone oxidoreductase which, contrary to what happens in aerobic conditions, reduces the rhodoquinone (RQ) instead of reducing the ubiquinone (UQ). This transfer of electrons is favorable because RQ has a redox potential of *E*_m_′ = −63 mV, which is lower than that of UQ (*E*_m_′ = +110 mV) [106,107]. The RQ donates electrons to the FDR to generate succinate from fumarate [108]. The measurement of FDR activity [39,109] is indicative of anaerobic metabolism [47,99], whereas the measurement of cytochrome *c* oxidase activity, of SDH, as well as the sensitivity of the electron transport chain (ETC) to different inhibitors (such as cyanide), are indicative of aerobic metabolism [62,110]. One point to highlight is that when NADH-rhodoquinone oxidoreductase participates, protons are translocated from the mitochondrial matrix to the intermembrane space, which, in turn, maintains a chemiosmotic gradient and generates ATP even in the absence of O_2_ (Figure 4).

In anaerobic metabolism [39], FRD performs the reverse reaction of succinate dehydrogenase (SDH) [40]. Both enzymes, SDH and FRD, are heterotetramers that share: (a) subunit 1 (Fp) that contains flavin-adenine dinucleotide (FAD); (b) subunit 2 (Ip) with three Fe-S centers; and (c) two subunits CybL and CybS, which maintain, on the one hand, binding to the inner mitochondrial membrane and, on the other, binding to the corresponding quinone [111,112]. In *A. suum*, there are isoforms in two of the four subunits as well; Fp and CybS are different between the aerobic larva and the anaerobic adult; no isoforms have been reported for the Ip and CybL subunits [113].

Considering the above, we can note that anaerobiosis-specific reactions are those catalyzed by FRD and ASCT. However, it is the presence of RQ that appears to be the only real difference between aerobic and anaerobic energy metabolism [114], as FRD expression has been described in cancer cells [115,116], while ASCT is an enzyme homologous to other transferases [105,117,118].

To recapitulate, in the cytosol of muscle cells under hypoxic conditions, lactate is produced by lactic acid fermentation. Unlike this, malate dismutation or malic fermentation has the following relevant aspects:

- It occurs in two cell compartments: cytosol and mitochondrion
- It is coupled to the generation of the proton gradient produced in complex I of the ETC, when the NADH coming from the formation of Acetyl-CoA and the one resulting from the activity of mME are oxidized (Figure 1).
- ATP is produced in the absence of O_2_ since the redox potential difference between NAD^+^/NADH (*E*_m_′ = −320 mV) and fumarate/succinate (*E*_m_′ = +30 mV) is sufficient for its synthesis [57,63]
- At the end of the pathway, several reduced compounds are obtained, including succinate, acetate, and propionate [119].

However, both aerobic and anaerobic metabolisms have the following aspects in common:

- The need for a final electron acceptor molecule
- Maintenance of redox balance
- Both are carried out in the mitochondrial compartments, which allows the formation of a proton gradient and therefore the synthesis of ATP.

Regardless of the type of energy metabolism, the redox balance is maintained. To keep it, organisms recycle their electron transporting coenzymes; thus, the number of reactions that produce NADH is equal to the reactions that consume it, or else, electrons are excreted in form to water, in aerobic organisms, and through succinate mainly in anaerobes [9].

### 3.3. Molecular Adaptations

The parasite-host relationship is, in most cases, a reciprocal interaction, in which the behavior of the parasite causes feedback in the host and vice versa. Because of this, analyzing this type of feedback mechanism is essential to understand the complex connections between animal behavior, ecology, and parasite evolution [120]. Up to this point, we can reflect on the convenience of using fermentative pathways in the maintenance of parasitic platyhelminths when aerobic respiration (consequently, settling in a place where an abundant supply of oxygen is available) would result in a greater supply of energy and, possibly, a higher metabolic rate. However, when dealing with parasitic forms, it is more important to establish the parasite in a strategic ecological niche that ensures a constant supply of substrates coming from the host (such as liver tissues in the case of flukes or the duodenum in the case of cestodes). Consequently, by having the resources secured, the parasites will concentrate on managing them [121].

One consequence of the foregoing is the parasite’s need to interact with its host and be able to maintain its ecological niche by engaging in chemical communication with it. It is not surprising then that the parasite has developed and fine-tuned mechanisms to evade the immune response. For example, it is known that some adult schistosomes can live from three to 10 years in humans, despite the harsh intravascular environment and their constant exposure to the immune system [46]. In fact, it has been possible to verify a registry of patients infected with *S. mansoni* for more than 30 years and the case of a patient infected with *E. granulosus* for 53 years [121].

The feedback between parasitic flatworms and their hosts has been studied using model organisms. For example, the murine experimental model of cysticercosis has made it possible to evaluate the interaction of the host (mice) with the cysticercus of *T. crassiceps* during its proliferation in the peritoneal cavity. This made it possible to describe some of these complex interactions [122], like the importance of the genetic factors of the host (the murine strain used) in the establishment and proliferation of the parasite. Another interesting observation is the importance of the sex of the host as it has been observed that cysticerci grow preferentially in female mice, regardless of the strain of *T. crassiceps*. Apparently, this sexual dimorphism is mediated by hormonal factors, since estrogens favor and androgens hinder the asexual reproduction of cysticerci [123]. Interestingly, when such cysticerci are inoculated into male mice, a feminization phenomenon can be observed in which testosterone levels decrease and estradiol levels increase [124]. The last section of this review will discuss parasite-host feedback in greater detail.

#### 3.3.1. Immune Response and Oxidative Stress in Parasitic Flatworms

##### Sources of Exposure to Reactive Oxygen Species

During establishment of the infection, the parasites induce a rapid immune response in the host, although it is nonspecific [125]. In general, this involves the activation of eosinophils, neutrophils, and macrophages, as well as the release of cytokines and the production of antibodies (IgE) [126]. These cells can produce large amounts of ROS and reactive nitrogen species (RNS), capable of directly destroying parasite cells. For example, liver flukes, such as *Opisthorchis viverrini*, induce chronic inflammation of the hepatobiliary system, exposing themselves to large amounts of ROS/RNS released by activated inflammatory cells [127]. Similarly, when an infection by the cestode *Taenia hydatigena* occurs in the peritoneum, an increase in the infiltration of small peritoneal macrophages responsible for a high production of nitric oxide (NO) can be observed, which harms the parasite and modulates the immune response [128]. However, the presence of immune cells induced by parasitic flatworms may be due to their participation in other processes such as wound repair caused by the migration of *F. hepatica* through the liver parenchyma [129].

**Figure 5:**
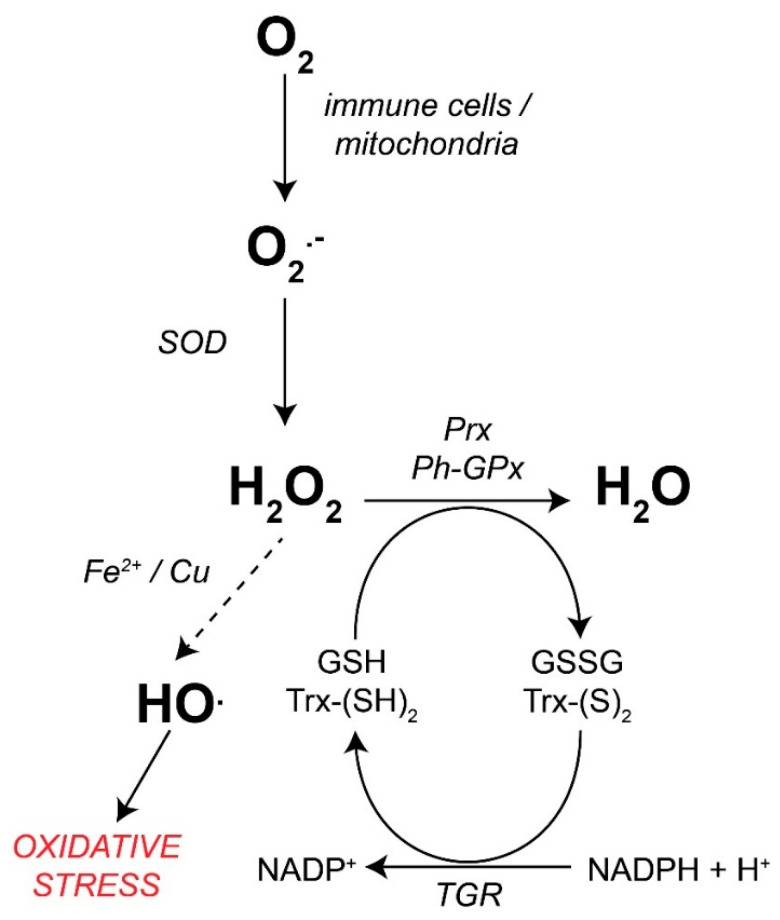
The antioxidant system of parasitic flatworms. *Abbreviations:* GSH reduced glutathione; GSSG, oxidized glutathione; H_2_O_2_, hydrogen peroxide; HO^●^, hydroxyl radical; O_2_, molecular oxygen; O_2_^●−^ superoxide anion radical; Ph-GPx, glutathione phospholipid peroxidase; Prx, peroxiredoxin; SOD, superoxide dismutase; TGR, thioredoxin-glutathione reductase; Trx-(SH)_2_, reduced thioredoxin; Trx-(S)_2_, oxidized thioredoxin.

The production of ROS/RNS due to the immune response has already been discussed in detail previously [130]. In general, the precursor of all ROS is the superoxide anion radical (O_2_^•−^), which is generated in leukocytes through the integral membrane enzyme NADPH oxidase (NOX), and by transferring an electron from NADPH to O_2_. O_2_^•−^ can undergo a spontaneous dismutation reaction generating hydrogen peroxide (H_2_O_2_) and O_2_. H_2_O_2_ can serve as a substrate for the enzyme myeloperoxidase (MPO) to generate the microbicidal compound hypochlorous acid (HClO). In the presence of transition elements such as ferrous (Fe^2+^) or copper (Cu^+^) ions, H_2_O_2_ can be reduced by the Fenton reaction, which produces the hydroxyl anion (HO^−^) and the hydroxyl radical (HO^•^). The HO^•^ radical is highly reactive, so it can subtract electrons from other biomolecules, like proteins, changing their properties and biological activities, with DNA generating mutations and membrane lipids initiating the lipid peroxidation process. This damage can lead to altered metabolism and eventually cell death (Figure 5).

It is important to clarify that another source of ROS is the metabolism of the parasites themselves, possibly due to their accelerated metabolism and the use of the malate dismutation pathway to obtain energy. It is in this pathway where the mitochondrial complex I continues to work, becoming an important place of ROS generation [131]. In mitochondria isolated from the tegument of *T. crassiceps*, a high production of H_2_O_2_ was recorded, unlike that observed in rat liver mitochondria. The high production of H_2_O_2_ associated with tegumental aerobic mitochondria has been observed with confocal microscopy in the cysticercus of *T. crassiceps* [94].

An important characteristic of these ROS and RNS is that they are short-lived intermediate products enzymatically synthesized by aerobic organisms and their clearance is regulated by enzymatic or non-enzymatic antioxidants. In this sense, there is a major co-evolutionary arms race competition between ROS production by the host and ROS scavenger by parasites; both closely related. For example, the production of H_2_O_2_ by hemocytes of the snail *Biomphalaria glabrata* when infected with *S. mansoni* sporocysts varies from population to population and in those snails with a high natural resistance against *S. mansoni* a higher production of H_2_O_2_ is observed, being preferentially infected by sporocysts with high levels of expression of their antioxidant systems [132].

##### Functioning and Localization of Enzymatic Antioxidant Systems in Parasitic Flatworms

(1) Superoxide Dismutase and Peroxidases

As mentioned before, HO^•^ is chemically very reactive, so the first line of defense of parasitic flatworms is to prevent its production, the controlled reduction of H_2_O_2_ to H_2_O being vital. The classic enzyme that carries out this reaction in aerobic organisms is catalase (CAT), which is absent in the genomes of parasitic flatworms [92,133] but is conserved in their free-living counterparts [134]. Hence, these parasites depend on enzymes glutathione peroxidase (GPx) and peroxiredoxin (Prx) in addition to the superoxide dismutase (SOD) necessary for O_2_^•−^ scavenging.

SOD is a family of metalloenzymes specialized in carrying out the dismutation of O_2_^•−^, generating O_2_ and H_2_O_2_. In animals, it is possible to identify two isoforms: (a) cytoplasmic SOD dependent on one atom of copper and another of Zn (Cu/Zn SOD); (b) mitochondrial SOD, dependent on a manganese atom (Mn SOD). Experimentally, it has been possible to isolate the enzyme [71] or clone the gene [135] from some parasitic flatworms, such as *T. solium*; thus, it was not surprising to confirm that, in genomes/transcriptomes, both trematode [125] and cestode [92,136] genes are present in both isoforms. Regarding their expression, their presence has been determined in all stages of the life cycle associated mainly with the tegument of these organisms [31], although a greater expression has been observed in the adult forms with respect to the larval [137].

GPx comprises isoenzymes that carry out the reduction of H_2_O_2_ to H_2_O, requiring electrons from two molecules of the tripeptide glutathione (GSH), taking it to its oxidized disulfide form (GSSG). Although this family has many representatives in mammals [138], in trematodes [125] and cestodes it has only been possible to identify a single gene [92] corresponding to a selenium-dependent GPx. This GPx type turned out to be membrane integral and it is exclusive for the reduction of lipid hydroperoxides (Ph-GPx); thus, it makes sense that it has been located in the tegument [137] of the different stages of trematodes *S. mansoni* [125] and *Fasciola gigantica* [139], and of the cestode *E. granulosus* [140]. Like SOD, this enzyme has a differential expression that is a function of the pO_2_ to which the parasite is exposed, finding its highest expression in the aerobic life cycle stages and its lowest in the anaerobic [141]. However, the overexpression of this enzyme responds to the exposure of exogenous oxidants, as shown in *C. sinensis* under in vitro conditions [142]. Cai et al., suggesting that Ph-GPx activity could be more focused on egg production than on the maintenance of the redox status [142].

Prxs are homodimeric proteins that catalyze the reduction of H_2_O_2_ and alkyl hydroperoxides to water and alcohol, respectively. The electrons that translocate frequently come from the 12 kDa protein thioredoxin (Trx), which is why these enzymes are also called thioredoxin peroxidases (TPx), although some isoforms can also obtain electrons from GSH. Despite the abundance of Prxs, their catalytic efficiency is lower than that of CAT or GPxs by one to three orders of magnitude [143]. However, this family of enzymes seems to be the most important in the H_2_O_2_ degradation process, both in trematodes and cestodes. In fact, in the genomes of *E. granulosus*, *E. multilocularis*, *H. microstoma*, and *T. solium*, it has been possible to identify three different genes encoding Prx 1–3 [92], which is consistent with the identification of three Prxs in *S. mansoni* [85]. Wang et al. reported that GPx activity in echinococcal cysts is practically undetectable, suggesting the relevance of Prxs in this parasite [144]. Using western blot analysis, it was found that in the trematode *O. viverrini*, OvTPx-1 is expressed in all stages of development; even if its location is different, depending on the isoenzyme [127]. For example, in adult flukes of *Schistosoma japonicum* it has been possible to locate Prx-1 in the tegument whereas Prx-2 has been found associated with the parenchyma, vitelline glands, and gastric epithelium [145]. In *H. diminuta*, a peroxidase-like activity was described in 1968 [146].

(2) Thioredoxin-Glutathione Reductase

To carry out peroxide hydrogen reduction, both the GPx and Prx (TPx) need to take electrons from GSH and Trx and then generate their oxidized forms, the substrates (i.e., GSSG and Trx-S_2_, respectively). Reductases responsible for reducing these substrates again and helping to maintain the homeostatic redox cycle are significantly necessary. In mammals, there are two enzymes called glutathione reductase (GR) and thioredoxin reductase (TrxR), both are NADPH-dependent for the reduction of GSSG and Trx-S_2_, respectively [147]. However, in 2001, Gladyshev et al. identified an enzyme capable of reducing both Trx-S_2_ and GSSG in the mouse testis. This enzyme was called thioredoxin-glutathione reductase (TGR) [148].

Although this enzyme has been identified in vertebrate organisms, including humans (hTGR) [149], it is in the group of parasitic flatworms where its study has gained relevance. Early on, it was isolated and characterized in the trematode *S. mansoni* (SmTGR) [150], as well as in the cestodes *E. granulosus* (EgTGR) [151] and *T. crassiceps* (TcTGR) [152]. Later on, it was possible to deduce its presence in other parasitic flatworms thanks to genomic advances [92]. Unlike their vertebrate hosts and their free-living counterparts [153], these organisms depend exclusively on this enzyme to carry out the reduction of GSSG and Trx-S_2_. Although it has been proposed that having an enzyme capable of reducing substrates belonging to two independent redox systems represents an evolutionary advantage [148], it is also possible to note that the dependence of parasitic flatworms on this enzyme makes it an excellent pharmacological target [154,155,156,157,158,159,160,161].

The genomes of trematodes and cestodes [76,87,92,129,162], as well as of the monogenean *Gyrodactylus salaris* [21], have corroborated the classic GRs and TrxRs in this group of parasites and have revealed that the TGR is encoded by a single gene; therefore, the cytosolic and mitochondrial forms must be generated by alternative splicing [163]. Despite being exactly of the same sequence, the environment in which it is located (either the cytosol or the mitochondrial matrix) affects its kinetic constants [164]. This enzyme is expressed in all stages of the life cycle, as reported in *S. mansoni* [125]. We previously reported that the in vivo inhibition of the TGR in *T. crassiceps* cysticerci is sufficient to compromise the viability of the parasite by altering its redox state and glutathione metabolism [165], which agrees with observations made when incubating schistosomula in the presence of anti TGR iRNA [166]. Due to its importance, TGR has been crystallized [167], promoting the search for drugs capable of inhibiting it [154,155,156,157,158,159,160,161].

(3) Glutathione-S-Transferase and Other Antioxidant Molecules

Glutathione-S-transferase (GST) is a family of highly conserved detoxifying enzymes that participate in the metabolism of many xenobiotics, although, in mammals, it has been found that it can participate in other relevant physiological processes such as the synthesis of leukotrienes, prostaglandins, and steroid hormones, as well as in amino acid catabolism and modulation of signaling processes [168]. GST can conjugate a wide range of substrates of an electrophilic nature, like the glutathione thiolate anion (GSH), to generate glutathionylated compounds, which are less reactive and more soluble and can be eliminated more efficiently by the cell. The enzyme can also detoxify by non-covalently binding to a series of hydrophobic ligands [169]. In the case of parasitic flatworms, it has been proposed that GSTs are essential for survival because they eliminate toxic and xenobiotic compounds derived endogenously or exogenously (generated or administered by the host) [170].

There are three families of GSTs with distinctive structural characteristics and different evolutionary origins: (a) cytosolic GSTs, (b) microsomal GSTs or also called MAPEG (Membrane associated proteins involved in eicosanoid and glutathione metabolism), and (c) kappa-class mitochondrial GSTs [171]. However, cytosolic GSTs have been the most studied, identifying up to seven different classes in mammals: alpha, mu, pi, sigma, theta, zeta, and omega [172]. Parkinson et al. reported the presence of two sigma-type cytosolic GSTs in the larval form of *E. granulosus*; one mu class and one microsomal GST [83]. However, an in-depth analysis of the *C. sinensis* trematode genome suggests the presence of 12 cytosolic GSTs distributed in the mu, sigma, zeta, and omega classes; in addition to mitochondrial GST and microsomal GST [173]. This agrees with findings in cestode genomes, where an important presence of 10 genes for cytosolic GSTs of the mu class have been reported, in addition to two GST genes of the sigma class and one of the MAPEG class [92]. Interestingly, Nguyen et al. reported the presence of a new type of cytosolic sigma GST in the metacestode of *T. solium* (TsMsGST), specifically expressed in the cytosol of the scolex tegument and susceptible to praziquantel (a drug used against neurocysticercosis, and which does not normally interact with sigma GSTs) [170]. Finally, Iriarte et al. analyzed the genomic information available from several flatworm representatives and discovered the potential absence of omega-class GST in cestodes, contrary to observations in other species of *Schistosoma* as the planarian *Schmidtea mediterranea* [174].

Regarding their location and expression in parasitic flatworms, GSTs present complex patterns and depend on both the species and the stage of the life cycle in which they are found. For example, Mei and Loverde reported that, regardless of the parasite stage, when analyzing the transcript levels of different antioxidant enzymes in *S. mansoni*, GST transcripts were 100-times more abundant than GPx transcripts and 10-times more than SOD isoforms. However, when enzymes were localized by immunofluorescence in the adult fluke, GST isoforms were restricted to a reduced subpopulation of parenchymal cells as well as to immature germ cells in both males and females. In contrast, SOD and GPX isoforms were localized in the tegument [137].

Another example is observed in the mRNA expression of the omega class GSTs of *C. sinensis* (CsGSTo 1 and 2). It begins with a growing pattern in juveniles of two to four weeks of age, but there is no expression in the metacercaria form and, in contrast, they are overexpressed in eggs [175]. This was contrasted with immunodetection techniques, locating CsGSTo in the egg, vitelline follicles, seminal receptacles, and testes. Because the expression of CsGSTo remains at high levels, regardless of environmental stimuli, the authors propose that the expression of these GSTs is conditioned by sexual reproduction within the host and that the abundance of CsGSTo in the egg is a preparation for the hostile conditions that the parasite will face when expelled from the definitive host.

Other Detoxifying Proteins That Have Been Reported in Parasitic Flatworms:

(a) *Cytochrome p450*. This cytochrome has monooxygenase activity, which allows it to oxidize multiple exogenous molecules and contributes to their detoxification. Both in the flukes of *S. mansoni* [176] and *Opisthorchis felineus* [177], as well as in the genomes of the cestodes, only one copy of the gene has been found [92].
(b) *Phytochelatin synthase (PCS)*. This enzyme works together with GST in the detoxification of xenobiotics and in the uptake of potentially harmful transition metals [178] through the formation of glutathione biopolymers [179]. Originally reported in plants, the presence of a functional PCS was reported in *S. mansoni* [180] and its presence was later confirmed in the genomes of cestodes [92] as well as in the parasitic nematode *Ancylostoma ceylanicum* [181]. Previously, we reported the presence of three unknown thiols in an extract of low molecular weight thiols obtained from the cysticercus of *T. crassiceps* [165] and whose retention patterns coincide with those found in *S. mansoni* and are associated with phytochelatins of different sizes [180]. Significantly, both phytochelatins and the PSC gene are absent in the mammalian hosts, suggesting that it is an adaptation to parasitic life [182], although its specific function is still under discussion [183].
(c) *Myoglobin (Mb)*. We have previously talked about the capacity of Mb to store O_2_. Other activities that Mb presents are peroxidase/dioxygenase, having the ability to interact with O_2_ molecules such as NO, CO, and H_2_O_2_ [184]. Ren et al. reported that a globin from *C. sinensis* (CsMb) showed peroxidase activity and that it may be important for ROS detoxification because of its overexpression after incubation with exogenous H_2_O_2_ [185]. This was later corroborated by Kim et al., who showed that incubation of *C. sinensis* flukes under aerobic conditions or in the presence of nitric oxide or nitrite is sufficient to induce the expression of the gene encoding CsMb [72]. Interestingly, overexpression of CsMb was also observed when flukes were co-incubated with human cholangiocytes (bile epithelial cells).
(d) *Other enzymes*. Under conditions of oxidative stress, hydroxyl groups can be nonspecifically oxidized to their aldehyde form. Similarly, reactions with radicals can lead to the formation of reactive carbonyls. As part of the characterization of the response of *E. granulosus* protoscolex to oxidative stress by exogenous H_2_O_2_, Cancela et al. reported high levels of a type of aldo-keto reductase (AKR), estradiol-17-beta dehydrogenase, and the enzyme carbonyl reductase 1 (CBR) [31]. AKRs are NADPH-dependent enzymes that can reduce aldehydes to alcohols [186]. On the other hand, CBR is an enzyme necessary to detoxify reactive carbonyls [187].
(4) Complexity of the Antioxidant Response

The different antioxidant enzymes do not work in isolation because to successfully face oxidative challenges, all systems must work together and simultaneously to avoid, as much as possible, the generation of highly toxic ROS such as the HO• radical. The latter maintains the peroxidases functioning by regenerating their electron source and repairing the damage caused during oxidative stress. In addition to this, there may be other factors that influence the antioxidant response, such as:

- *Time elapsed since the establishment of the infection.* Skrzycki et al. compared oxidative stress markers and the presence of antioxidant enzymes in two populations of the adult *H. diminuta* cestode, one with a short experimental infection time and another with a well-consolidated infection [126]. They found a high activity of the enzymes SOD, Ph-GPx, and Prx in the anterior end (close to the intestinal epithelium) comparable to that of both tapeworms. However, in older tapeworms they found higher GST activity and lower GSH concentration, which suggests that adults also face a constant detoxification process. As the tapeworm size increases and occupies the ileum, oxidant indicators increase with a progressive decrease in antioxidant enzymes (except GST); however, at the posterior end of the parasite, where the proglottids are sexually mature, antioxidant enzymes increase again. This suggests that the production and storage of eggs, which occurs in the mature and gravid proglottids located at the posterior end of tapeworms, requires the participation of antioxidant systems. In the case of old tapeworms, a similar pattern of antioxidant enzyme activity is observed, but contrary to expectations, oxidative stress markers always remained below the levels reached by their young counterparts. This suggests that by consolidating the infection, old tapeworms have managed to modulate the immune response, which leads to less exposure to ROS. Finally, the only enzyme that does not significantly reduce its activity is GST, which implies that the parasite is always ready to purge toxic metabolites.
- *Sexual dimorphism of the parasite.* Oliveira et al. compared the contribution of nutrients and gender of unpaired adults of *S. mansoni* on O_2_ consumption pathways and susceptibility to oxidative stress [85]. In general, they found a greater contribution of glutamine to respiration in females, which contrasts with a greater contribution of glucose in the case of males. The O_2_ consumption rate was higher in males compared to females, regardless of the respiratory substrate. In contrast, the rate of ROS production and the expression of antioxidant enzymes was higher in females than in males. This suggests that the physiological process of egg production is related to an increase in endogenous ROS. Finally, females were more tolerant to exogenous oxidative stress than males, possibly due to basal overexpression of their antioxidant systems.

#### 3.3.2. Parasite-Host Relationships

Parasitic platyhelminths establish a chemical dialogue with the host by taking elements from it and by sending molecules from the parasite, having a different impact in their relationship with the host. In addition to oxygen diffusion, this class of parasites must ensure access to the nutrients they need for their development, so the presence of a sophisticated recognition and acquisition system through the use of specific transporters (similar to those of the host) is not surprising [30,188]. However, the parasite-host relationship is not limited to that, as the parasite can use the metabolism of the host cells to its advantage [93],

Through proteomic analysis, the presence of intact and functional host proteins has been confirmed in the hydatid fluid of *E. granulosus* and in the vesicular fluid of various species of the genus *Taenia* [50]. Although the ratio of parasite/host proteins is specific to each organism, the composition of these fluids against the composition of the serum of the respective host has been analyzed [50,189,190,191,192]. Some of the most abundant host proteins reported are serum albumin and immunoglobulins [87,190]. In the case of the former, the parasite can use it to maintain internal osmotic pressure, whereas the latter could help to prevent antigen exposure of the immune system [192,193]. Surprisingly, it has been reported that these organisms can incorporate various host antioxidant proteins to their antioxidant repertoire, such as the SOD, Prxs, and CAT isoforms [81,87].

In parasitic flatworms, the presence of various families of transporters specialized in the removal of metabolites and drugs has been reported. Although this representation is not homogeneous in flatworms, its participation in detoxification processes has been demonstrated [194,195,196,197,198,199,200,201]. In a previous experiment, we inhibited the TGR enzyme activity of *T. crassiceps* cysticerci under in vitro conditions and observed the appearance of GSSG in the culture medium, which led to the proposition that the cestode expels GSSG excess as a mechanism to avoid the change of the redox environment inside [165]. By searching for a transporter capable of carrying out the translocation of this oxidized species, we were able to identify some multidrug resistance (MDR) transporters in the genome of *T. solium*, which may potentially be responsible for carrying out this function [165].

## 4. Conclusions

Oxygen has a dual function in organisms. In aerobic organisms it works mainly as a final electron acceptor during respiration, which results in greater energy production through the catabolic pathways, and its presence is related to the generation of ROS resulting in the expression of antioxidant systems involved in redox homeostasis maintenance.

Among flatworms, trematodes and cestodes have life cycles that develop in environments with different oxygen tension, which determine the development of special characteristics that have allowed them to adapt to varied conditions, such as:

- An energy metabolism that transits between aerobiosis and anaerobiosis depending on the availability of oxygen.
- This is possible because they have an enzymatic repertoire with common metabolic pathways involving enzymes that catalyze reversible reactions.
- In anaerobiosis, in addition to lactic fermentation, they have another fermentation pathway known as malate dismutation that allows them to obtain a greater amount of energy even in the absence of oxygen. Additionally, this pathway allows them to maintain their redox balance by eliminating the electrons in molecules that are secreted into the medium, mainly succinate, acetate, and propionate.
- Due to the absence of a circulatory system, they developed a tegument through which O_2_ diffuses.
- The diffusion of oxygen generates the formation of a concentration gradient, its presence being greater in the tegument than in the parenchyma.
- Two populations of mitochondria, aerobic and anaerobic, have been described; the first located mainly in the tegument.
- Finally, the exposure of the tegument to a higher concentration of O_2_ implies a greater production of ROS in it, as indirectly demonstrated by a significant presence of antioxidant enzymes in this region (SOD, GPx, Prx).

These overall data provide more information on the type of metabolism that is performed in the parasite in relation to pO_2_. However, as Boyunaga comments [82], “one must be cautious when trying to relate this O_2_ tension where these parasites develop” with the type of energy metabolism they carry out, since the reports in the literature can be controversial. Thus, an important aspect that must be considered is the presence of both types of metabolism, aerobic and anaerobic, in the same organism and its relation to the size of the parasite, the stage of the life cycle, and the degree of purity of the mitochondrion (at least two mitochondrial types in these organisms). Having pure populations of mitochondria would make it possible to determine with greater certainty what type of energy metabolism occurs at what time in the life cycle and in what region of the parasite.

## Figures and Tables

**Table 1:** Oxygen concentration during the life cycle of *Taenia solium*.

Specie	Biologic Form	Host	Localization in Host	Oxygen Concentration		References
				pO_2_	mmHg	
*T. solium*	Egg	Enviroment	NA	21.1	160	[33]
	Oncosphera	Pig	Duodenum	5.9	45	[34]
			Blood capillaries *	5.3–13.2	40–100	[33]
	Cysticerci		Muscle	4.9	37.5	[34]
			Brain	3.9	30	[34]
	Taenia	Human	Duodenum **	7.9	60	[35]
			Ileum **	1.3	10	[35]
			Cecum **	0	0	[35]
	Cysticerci		Muscle (in rest)	3.6–3.9	27–30	[36]
			Brain (grey matter)	2.1–5.3	16–40	[36]
			Brain (white matter)	3.2–4.4	24–33	[36]

* Human measurements. ** Mice measurements.

## References

[1] Hamilton T.L., Bryant D.A., Macalady J.L. (2016). The role of biology in planetary evolution: Cyanobacterial primary production in low-oxygen Proterozoic oceans. Environ. Microbiol..

[2] Fisher W.W., Hemp J., Valentine J.S. (2016). How did life survive earth’s great oxygenation?. Curr. Opin. Chem. Biol..

[3] Lenton T.M., Dahl T.W., Daines S.J., Mills B.J.W., Ozaki K., Saltzman M.R., Porada P. (2016). Earliest land plants created modern levels of atmospheric oxygen. Proc. Natl. Acad. Sci. USA.

[4] Morris J.L., Puttick M.N., Clark J.D., Donoghue P.C.J. (2018). The timescale of early land plant evolution. Proc. Natl. Acad. Sci. USA.

[5] Lyons T.W., Reinhard C.T., Planavsky N.J. (2014). The rise of oxygen in Earth´s ocean and atmosphere. Nature.

[6] Zimorski V., Mental M., Tielens A.G.M., Martin W.F. (2019). Energy metabolism in anaerobic eukaryotes and Earth’s late oxygenation. Free Radic. Biol. Med..

[7] Mentel M., Martin W. (2008). Energy metabolism among eukaryotic anaerobes in light of Proterozoic ocean chemistry. Phil. Trans. R. Soc. B Biol. Sci..

[8] Mentel M., Rottger M., Leys S., Tielens A.G.M., Martin W.F. (2014). Of early animals, anaerobic mitochondria, and a modern sponge. Bioassays.

[9] Muller M. (2012). Biochemistry and evolution of anaerobic energy metabolism in eukaryotes. Microbiol. Mol. Biol. Rev..

[10] Tielens A.G.M., Rotte C., van Hellemond J.J., Martin W. (2002). Mitochondria as we don’t know them. Trends Biochem. Sci..

[11] Meléndez-Hevia E., Montero-Gómez N., Montero F. (2008). From prebiotic chemistry to cellular metabolism-The chemical evolution of metabolism before Darwinian natural selection. J. Theor. Biol..

[12] Romano A.H., Conway T. (1996). Evolution of carbohydrate metabolic pathways. Res. Microbiol..

[13] Van Der Giezen M., Lenton T.M. (2012). The rise of oxygen and complex life. J. Eukaryot. Microbiol..

[14] Halliwell B., Gutteridge J.M. (1984). Oxygen toxicity, oxygen radicals, transition metals and disease. Biochem. J..

[15] Sawyer D.T., Valentine J.S. (1981). How Super is Superoxide?. Acc. Chem. Res..

[16] Collins J.J. (2017). Platyhelminthes. Curr. Biol..

[17] Bobes R.J., Fragoso G., Fleury A., García-Varela M., Sciutto E., Larralde C., Laclette J.P. (2014). Evolution, molecular epidemiology and perspectives on the research of taeniid parasites with special emphasis on *Taenia solium*. Infect. Genet. Evol..

[18] Egger B., Steinke D., Tarui H., De Mulder K., Arendt D., Borgonie G., Funayama N., Gschwentner R., Hartenstein V., Hobmayer B. (2009). To be or not to be a flatworm: The acoel controversy. PLoS ONE.

[19] Riutort M., Álvarez-Presas M., Lázaro E., Solà E., Paps J. (2012). Evolutionary history of the Tricladida and the Platyhelminthes: An up-to-date phylogenetic and systematic account. Int. J. Dev. Biol..

[20] Cheng L.C., Tu K.C., Seidel C.W., Robb S., Guo F., Sánchez Alvarado A. (2018). Cellular, ultrastructural and molecular analyses of epidermal cell development in the planarian Schmidtea mediterranea. Dev. Biol..

[21] Hahn C., Fromm B., Bachmann L. (2014). Comparative genomics of flatworms (platyhelminthes) reveals shared genomic features of ecto- and endoparastic neodermata. Genome Biol. Evol..

[22] Perkins E.M., Donnellan S.C., Bertozzi T., Whittington I.D. (2010). Closing the mitochondrial circle on paraphyly of the Monogenea (Platyhelminthes) infers evolution in the diet of parasitic flatworms. Int. J. Parasitol..

[23] Harrington D., Lamberton P., McGregor A. (2017). Human liver flukes. Lancet Gastroenterol. Hepatol..

[24] Toledo A., Osorio R., Matus C., Martinez Lopez Y., Ramirez Cruz N., Sciutto E., Fragoso G., Arauz A., Carrillo-Mezo R., Fleury A. (2018). Human Extraparenchymal Neurocysticercosis: The Control of Inflammation Favors the Host…but Also the Parasite. Front. Immunol..

[25] Hayward A.D., Skuce P.J., McNeilly T.N. (2021). The influence of liver fluke infection on production in sheep and cattle: A meta-analysis. Int. J. Parasitol..

[26] Cwiklinski K., O’Neill S.M., Donnelly S., Dalton J.P. (2016). A prospective view of animal and human Fasciolosis. Parasite Immunol..

[27] Trevisan C., Devleesschauwer B., Schmidt V., Winkler A.S., Harrison W., Johansen M.V. (2017). The societal cost of *Taenia solium* cysticercosis in Tanzania. Acta Trop..

[28] Neves L.X., Wilson R.A., Brownridge P., Harman V.M., Holman S.W., Beynon R.J., Eyers C.E., DeMarco R., Castro-Borges W. (2020). Quantitative Proteomics of Enriched Esophageal and Gut Tissues from the Human Blood Fluke *Schistosoma mansoni* Pinpoints Secreted Proteins for Vaccine Development. J. Proteome Res..

[29] Morley N.J. (2015). Ecology of free-living metacercariae (Trematoda). Adv. Parasitol..

[30] Li W.H., Yang Y., Zhang N.Z., Wang J.K., Liu Y.J., Li L., Yan H.B., Jia W.Z., Fu B. (2021). Comparative Transcriptome Analyses of the Developmental Stages of *Taenia multiceps*. Front. Vet. Sci..

[31] Cancela M., Paes J.A., Moura H., Barr J.R., Zaha A., Ferreira H.B. (2019). Unraveling oxidative stress response in the cestode parasite Echinococcus granulosus. Sci. Rep..

[32] Bryant C., Behm C.A., Van den Bossche H. (1976). Biochemistry of Parasites and Host Parasite Relationships.

[33] Carreau A., El Hafny-Rahbi B., Matejuk A., Grillon C., Kieda C. (2011). Why is the partial oxygen pressure of human tissues a crucial parameter? Small molecules and hypoxia. J. Cell Mol. Med..

[34] De Santis V., Singer M. (2015). Tissue oxygen tension monitoring of organ perfusion: Rationale, methodologies, and literature review. Br. J. Anaesth..

[35] Friedman E.S., Bittinger K., Esipova T.V., Hou L., Chau L., Jiang J., Mesaros C., Lund P.J., Liang X., FitzGerald G.A. (2018). Microbes vs. chemistry in the origin of the anaerobic gut lumen. Proc. Natl. Acad. Sci. USA.

[36] Mori M.P., Penjweini R., Knutson J.R., Wang P., Hwang P.M. (2021). Mitochondria and oxygen homeostasis. FEBS J..

[37] Komuniecki R., Tielens A.G.M., Marr J.J., Nilsen T.W., Komuniecki R.W. (2003). Carbohydrate and energy metabolism helminths. Molecular Medical Parasitology.

[38] Ward J.B.J., Keely S.J., Keely S.J. (2014). Oxygen in the regulation of intestinal epithelial transport. J. Phisiol..

[39] Harada S., Inaoka D.K., Ohmori J., Kita K. (2013). Diversity of parasite complex II. BBA.

[40] Van Hellemond J.J., Tielens A.G.M. (1994). Expression and functional properties of fumarate reductase. Biochem. J..

[41] Kita K., Nihei C., Tomitsuka E. (2003). Parasite Mitochondria as Drug Target: Diversity and Dynamic Changes During the Life Cycle. Curr. Med. Chem..

[42] Tielens A.G.M., van den Heuvel J.M., van den Berg S.G. (1987). Differences in intermediary energy metabolism between juvenile and adult *Fasciola hepatica*. Mol. Biochem. Parasitol..

[43] Tielens A.G.M. (2000). The carbohydrate metabolism of *Fasciola hepatica*, an example of biochemical adaptations in parasitic helminths. Acta Parasitol..

[44] Poddubnaya L.G., Scholz T., Kuchta R., Levron C., Brunanská M. (2007). Ultrastructure of the proglottid tegument (neodermis) of the cestode *Echinophallus wageneri* (Pseudophyllidea: Echinophallidae), a parasite of the bathypelagic fish *Centrolophus niger*. Parasitol. Res..

[45] Wendt G.R., Collins J.N., Pei J., Pearson M.S., Bennett H.M., Loukas A., Berriman M., Grishin N.V., Collins J.J. (2018). Flatworm-specific transcriptional regulators promote the specification of tegumental progenitors in *Schistosoma mansoni*. Elife.

[46] Sotillo J., Pearson M., Becker L., Mulvenna J., Loukas A. (2015). A quantitative proteomic analysis of the tegumental proteins from *Schistosoma mansoni* schistosomula reveals novel potential therapeutic targets. Int. J. Parasitol..

[47] Takamiya S., Fukuda K., Nakamura T., Aoki T., Sugiyama H. (2010). *Paragonimus westermani* possesses aerobic and anaerobic mitochondria in different tissues, adapting to fluctuating oxygen tension in microaerobic habitats. Int. J. Parasitol..

[48] Tielens A.G.M., van den Heuvel J.M., van den Bergh S.G. (1984). The energy metabolism of *Fasciola hepatica* during its development in the final host. Mol. Biochem. Parasitol..

[49] Starling J.A. (1975). Tegumental carbohydrate transport in intestinal helminths: Correlation between mechanisms of membrane transport and the biochemical environment of absorptive surfaces. Trends Am. Microsc. Soc..

[50] Brehm K., Koziol U. (2017). Echinococcus-Host Interactions at Cellular and Molecular Levels. Adv. Parasitol..

[51] Van Hellemond J.J., Retra K., Brouwers J.F.H.M., van Balkom M.Y., Shoemarker C.B., Tielens A.G.T. (2006). Functions of the tegument of schistosomes: Clues from the proteome and lipidome. Int. J. Parasitol..

[52] Leow C.Y., Willis C., Hofmann A., Jones M.K. (2015). Structure-function analysis of apical membrane-associated molecules of the tegument of schistosome parasites of humans: Prospects for identification of novel targets for parasite control. Br. J. Pharmacol..

[53] Tovar J., Fischer A., Clark C.G. (1999). The mitosome, a novel organelle related to mitochondria in the amitochondrial parasite *Entamoeba histolytica*. Mol. Microbiol..

[54] Makiuchi T., Nozaki T. (2014). Highly divergent mitochondrion-related organelles in anaerobic parasitic protozoa. Biochimie.

[55] Palade G.E. (1953). An electron microscope study of the mitochondrial structure. J. Histochem. Chem. Cytochem..

[56] Scheffler I.E. (2011). Structure and morphology. Integration into the cell. Mitochondria.

[57] Kita K., Takamiya S. (2002). Electron-transfer complexes in *Ascaris* mitochondria. Adv. Parasitol..

[58] Semenza G.L. (2012). Hypoxia-inducible factors in physiology and medicine. Cell.

[59] Thompson D.P., Geary T.G., Marr J.J., Müller M. (2003). The Structure and Function of Helminth Surfaces: Structural. Biochemistry and Molecular Biology of Parasites.

[60] Tielens A.G.M. (1994). Energy generation in parasitic helminths. Parasitol. Today.

[61] Lumsden R.D. (1967). Ultrastructure of mitochondria in a cestode, *Lacistorrhynchus tenuis* (V. Benden, 1858). J. Parasitol..

[62] Del Arenal M.I.P., Cea B.A., Moreno-Sanchez R., Escamilla J.E. (1998). A method for the isolation of tegument syncytium mitochondria from *Taenia crassiceps* cysticerci and partial characterization of their aerobic metabolism. J. Parasitol..

[63] Takamiya S., Wang H., Hiraishi A., Yu Y., Hamajima F. (1994). Respiratory chain of the lung *Paragonimus westermani*: Facultative anaerobic mitochondria. Arch. Biochem. Biophys..

[64] Roppongi T., Mizuno N., Miyagawa Y., Kobayashi T., Nakagawa K., Adachi S. (2021). Solubility and mass transfer coefficient of oxygen through gas- and water-lipid interfaces. J. Food Sci..

[65] Terwilliger N.B. (1998). Functional adaptations of oxygen-transport proteins. J. Exp. Biol..

[66] Gell D.A. (2018). Structure and function of haemoglobins. Blood Cells Mol. Dis..

[67] Storz J.F., Opazo J.C., Hoffmann F.G. (2013). Gene duplication, genome duplication, and the functional diversification of vertebrate globins. Mol. Phylogenet. Evol..

[68] De Guzman J.V., Yu H.S., Jeong H.J., Hong Y.C., Kim J., Kong H.H., Chung D.I. (2007). Molecular characterization of two myoglobins of *Paragonimus westermani*. J. Parasitol..

[69] Goldberg D.E. (1995). The enigmatic oxygen-avid hemoglobin of *Ascaris*. Bioessays.

[70] Kiger L., Rashid A.K., Griffon N., Haque M., Moens L., Gibson Q.H., Poyart C., Marden M.C. (1998). Trematode hemoglobins show exceptionally high oxygen affinity. Biophys. J..

[71] González R., Mendoza-Hernández G., Plancarte A. (2002). Purification of *Taenia solium* cysticerci superoxide dismutase and myoglobin copurification. Parasitol. Res..

[72] Kim S.H., Yang D., Bae Y.A. (2021). Hypoxic and nitrosative stress conditions modulate expression of myoglobin genes in a carcinogenic hepatobiliary trematode, *Clonorchis sinensis*. PLoS Negl. Trop. Dis..

[73] Burmester T., Hankeln T. (2014). Function and evolution of vertebrate globins. Acta Physiol..

[74] McManus D.P. (1987). Intermediary metabolism in parasitic helminths. Int. J. Parasitol..

[75] Tielens A.G.M., van de Pas F.A., van den Heuvel J.M., van den Bergh S.G. (1991). The aerobic energy metabolism of *Schistosoma mansoni* miracidia. Mol. Biochem. Parasitol..

[76] Young N.D., Nagarajan N., Lin S.J., Korhonen P.K., Jex A.R., Hall R.S., Safavi-Hemami H., Kaewkong W., Bertrand D., Gao S. (2014). The *Opisthorchis viverrini* genome provides insights into life in the bile duct. Nat. Commun..

[77] Bertout J.A., Patel S.A., Simon M.C. (2008). The impact of O2 availability on human cancer. Nat. Rev. Cancer..

[78] Rytkonen K.T., Storz J.F. (2011). Evolutionary origins of oxygen sensing in animals. EMBO Rep..

[79] Goto M., Amino H., Nakajima M., Tsuji N., Sakamoto K., Kita K. (2013). Cloning and characterization of hypoxia-inducible factor-1 subunits from Ascaris suum-a parasitic nematode highly adapted to changes of oxygen conditions during its life cycle. Gene.

[80] Kim S.H., Oh G.S., Sohn W.M., Lee K., Yang H.J., Bae Y.A. (2019). Molecular characteristics and induction profiles of hypoxia-inducible factor-1α and other basic helix-loop-helix and Per-Arnt-Sim domain-containing proteins identified in a carcinogenic liver fluke *Clonorchis sinensis*. Parasitology.

[81] Cui S.J., Xu L.L., Zhang T., Xu M., Yao J., Fang C.Y., Feng Z., Yang P.Y., Hu W., Liu F. (2013). Proteomic characterization of larval and adult developmental stages in *Echinococcus granulosus* reveals novel insight into host-parasite interactions. J. Proteom..

[82] Boyunaga H., Schmitz M.G., Brouwers J.F., van Hellemond J.J., Tielens A.G.M. (2001). *Fasciola hepatica* miracidia are dependent on respiration and endogenous glycogen degradations for their energy generation. Parasitology.

[83] Parkinson J., Wasmuth J.D., Salinas G., Bizarro C.V., Sanford C., Berriman M., Ferreira H.B., Zaha A., Blaxter M.L., Maizels R.M. (2012). A transcriptomic analysis of *Echinococcus granulosus* larval stages: Implications for parasite biology and host adaptation. PLoS Negl. Trop. Dis..

[84] De Almeida Leandro L., Fraga C.M., de Souza Lino R., Vinaud M.C. (2014). Partial reverse of the TCA cycle is enhanced in *Taenia crassiceps* experimental neurocysticercosis after in vivo treatment with anthelminthic drugs. Parasitol. Res..

[85] Oliveira M.P., Correa Soares J.B., Oliveira M.F. (2016). Sexual Preferences in Nutrient Utilization Regulate Oxygen Consumption and Reactive Oxygen Species Generation in *Schistosoma mansoni*: Potential Implications for Parasite Redox Biology. PLoS ONE.

[86] Ritler D., Rufener R., Li J.V., Kämpfer U., Müller J., Bühr C., Schürch S., Lundström-Stadelmann B. (2019). In vitro metabolomic footprint of the *Echinococcus multilocularis* metacestode. Sci. Rep..

[87] Zhang S. (2019). Comparative Transcriptomic Analysis of the Larval and Adult Stages of *Taenia pisiformis*. Genes.

[88] Willms K., Robert L., Caro J.A. (2003). Ultrastructure of smooth muscle, gap junctions and glycogen distribution in *Taenia solium* tapeworms from experimentally infected hamsters. Parasitol. Res..

[89] Valkounová J., Zdárská Z., Slais J. (1992). Histochemistry of the racemose form of *Cysticercus cellulosae*. Folia Parasitol..

[90] Chekulayev V., Mado K., Shevchuk I., Koit A., Kaldma A., Klepinin A., Timohhina N., Tepp K., Kandashvili M., Ounpuu L. (2015). Metabolic remodeling in human colorectal cancer and surrounding tissues: Alterations in regulation of mitochondrial respiration and metabolic fluxes. Biochem. Biophys. Rep..

[91] Skelly P.J., Shoemaker C.B. (1995). A molecular genetic study of the variations in metabolic function during schistosome development. Mem. Ins. Oswaldo Cruz..

[92] Tsai I.J., Zarowiecki M., Holroyd N., Garciarrubio A., Sánchez-Flores A., Brooks K.L., Tracey A., Bobes R.J., Fragoso G., Sciutto E. (2013). The genomes of four tapeworm species reveal adaptations to parasitism. Nature.

[93] Fraga C.M., Costa T.L., Bezerra J.C., de Souza Lino R., Vinaud M.C. (2012). *Taenia crassiceps*: Host treatment alters glycolisis and tricarboxilic acid cycle in cysticerci. Exp. Parasitol..

[94] Del Arenal I.P., Rubio M.E., Ramírez J., Rendón J.L., Escamilla J.E. (2005). Cyanide-resistant respiration in *Taenia crassiceps* metacestode (cysticerci) is explained by the H_2_O_2_-producing side-reaction of respiratory complex I with O_2_. Parasitol. Int..

[95] Bennet E.M., Behm C.A., Bryant C. (1990). The role of the host in the regulation of end-product formation in two strains of the rat tapeworm, *Hymenolepis diminuta*. Int. J. Parasitol..

[96] Bryant C. (1993). Organic acid excretion by helminths. Parasitol. Today.

[97] Campbell T., Rubin N., Komuniecki R. (1989). Succinate-dependent energy generation in *Ascaris suum* mitochondria. Mol. Biochem. Parasitol..

[98] Tielens A.G.M., van Hellemond J.J. (1998). The electron transport chain in anaerobically functioning eukaryotes. Biochim. Biophys. Acta.

[99] Matsumoto J., Sakamoto K., Shinjyo N., Kido Y., Yamamoto N., Yagi K., Miyoshi H., Nonaka N., Katakura K., Kita K. (2008). Anaerobic NADH-fumarate reductase system is predominant in the respiratory chain of *Echinococcus multilocularis*, providing a novel target for the chemotherapy of Alveolar Echinococcosis. Antimicrob. Agents Chemother..

[100] Ovington K.S., Bryant C. (1981). The role of carbon dioxide in the formation of end-products *Hymenolepis diminuta*. Int. J. Parasitol..

[101] Fioravanti C.F., Vandock K.P. (2010). Transhydrogenase and the anaerobic mitochondrial metabolism of adult *Hymenolepis diminuta*. Parasitology.

[102] Van Hellemond J.J., van der Klei A., van Weelden S.W.H., Tielens A.G.M. (2003). Biochemical and evolutionary aspects of anaerobically functioning mitochondria. Philos. Trans. R. Soc. B Biol. Sci..

[103] Lima N.F., Picanço G.A., Costa T.L., de Souza Lino Junior R., Vinaud M.C. (2021). In Vivo Treatment with the Combination of Nitazoxanide and Flubendazole Induces Gluconeogenesis and Protein Catabolism in *Taenia crassiceps* cysticerci. Acta Parasitol..

[104] Tielens A.G.M., van Grinsven K., Henze K., van Hellemond J.J., Martin W. (2010). Acetate formation in the energy metabolism of parasitic helminths and protists. Int. J. Parasitol..

[105] Van Grinsven K.W.A., van Hellemond J.J., Tielens A.G.M. (2009). Acetate: Succinate CoA-transferase in the anaerobic mitochondria of *Fasciola hepatica*. Mol. Biochem. Parasitol..

[106] Moore H.W., Folkers K., Coenzyme Q. (1965). LXII. Structure and Synthesis of Rhodoquinone, a Natural Aminoquinone of the Coenzyme Q Group. J. Am. Chem. Soc..

[107] Van Hellemond J.J., Klockiewicz M., Gaasenbeek C.P., Roos M.H., Tielens A.G.M. (1995). Rhodoquinone and complex II of the electron transport chain in anaerobically functioning eukaryotes. J. Biol. Chem..

[108] Fioravanti C.F., Kim Y. (1988). Rhodoquinone requirement of the *Hymenolepis diminuta* mitochondrial electron transport system. Mol. Biochem. Parasitol..

[109] Boveris A., Hertig C., Turrens J. (1986). Fumarate reductase and other mitochondrial activities in *trypanosoma cruzi*. Mol. Biochem. Parasitol..

[110] Arrigoni O., Singer T.P. (1992). Limitations of the phenazine methosulfate assay for succinic and related dehydrogenases. Nature.

[111] Kuramochi T., Hirawake H., Kojima S., Takamiya S., Furushima R., Aoki T., Komuniecki R., Kita K. (1994). Sequence comparison between the flavoprotein subunit of the fumarate reductase (complex II) of the anaerobic parasitic nematode, *Ascaris suum* and the succinate dehydrogenase of the aerobic, free-living nematode, *Caenorhabditis elegans*. Mol. Biol. Parasitol..

[112] Amino H., Osanai A., Miyadera H., Shinjyo N., Tomitsuka E., Taka H., Mineki R., Murayama K., Takamiya S., Aoki T. (2003). Isolation and characterization of the stage-specific cytochrome b small subunit (CybS) of *Ascaris suum* complex II from the aerobic respiratory chain of larval mitochondria. Mol. Biochem. Parasitol..

[113] Amino H., Wang H., Hirawake H., Saruta F., Mizuchi D., Mineki R., Shindo N., Murayama K., Takamiya S., Aoki T. (2000). Stage specific isoforms of *Ascaris suum* complex II: The fumarate reductase of the parasitic adult and the succinate dehydrogenase of free-living larvae share a common iron-sulfur subunit. Mol. Biochem. Parasitol..

[114] Salinas G., Langelaan D.N., Shepherd J.N. (2020). Rhodoquinone in bacteria and animals: Two distinct pathways for biosynthesis of this key electron transporter used in anaerobic bioenergetics. BBA Bioenerg..

[115] Sakai C., Tomitsuka E., Esumi H., Harada S., Kita K. (2012). Mitochondrial fumarate reductase as a target of chemotherapy: From parasites to cancer cells. BBA.

[116] Tomitsuka E., Kita K., Esumi H. (2012). An anticancer agent, pyrvinium pamoate inhibits the NADH-fumarate reductase system-a unique mitochondrial energy metabolism in tumor microenvironments. J. Biochem..

[117] Saz H.J., deBruyn B., de Mata Z. (1996). Acyl-CoA transferase activities in homogenates of *Fasciola hepatica* adults. J. Parasitol..

[118] Rivière L., van Weelden S.W., Glass P., Vegh P., Coustou V., Biran M., van Hellemond J.J., Bringaud F., Tielens A.G.M., Boshart M. (2004). Acetyl: Succinate CoA-transferase in procyclic *Trypanosoma brucei*. Gene identification and role in carbohydrate metabolism. J. Biol. Chem..

[119] Fraga C.M., De Castro A.M., Reynoso-Ducoing O., Ambrosio J., Hernández-Campos A., Castillo R., Vinaud M.C. (2016). Alternative energy production pathways in *Taenia crassiceps* in vitro exposed to a benzimidazole derivative (RCB20). Parasitology.

[120] Ezenwa V.O., Archie E.A., Craft M.E., Hawley D.M., Martin L.B., Moore J., White L. (2016). Host behaviour-parasite feedback: An essential link between animal behaviour and disease ecology. Proc. Biol. Sci..

[121] Zarowiecki M., Berriman M. (2015). What helminth genomes have taught us about parasite evolution. Parasitology.

[122] Fragoso G., Bobes R.J., Espinoza B., Martínez M.L., Pérez-Morales D., Rosas G., Sciutto E., Laclette J.P. (2012). Changes in cyst’s nuclear chromatin resulting after experimental manipulation of *Taenia crassiceps* mice infections: Biological implications. Exp. Parasitol..

[123] Escobedo G., Larralde C., Chavarria A., Cerbón M.A., Morales-Montor J. (2004). Molecular mechanisms involved in the differential effects of sex steroids on the reproduction and infectivity of *Taenia crassiceps*. J. Parasitol..

[124] Larralde C., Morales J., Terrazas I., Govezensky T., Romano M.C. (1995). Sex hormone changes induced by the parasite lead to feminization of the male host in murine *Taenia crassiceps* cysticercosis. J. Steroid Biochem. Mol. Biol..

[125] Mourão M., Dinguirard N., Franco G.R., Yoshino T.P. (2009). Role of the endogenous antioxidant system in the protection of *Schistosoma mansoni* primary sporocysts against exogenous oxidative stress. PLoS Negl. Trop. Dis..

[126] Skrzycki M., Majewska M., Podsiad M., Czeczot H., Salamatin R., Twarowska J., Grytner-Zięcina B. (2011). *Hymenolepis diminuta*: Experimental studies on the antioxidant system with short and long term infection periods in the rats. Exp. Parasitol..

[127] Suttiprapa S., Sotillo J., Smout M., Suyapoh W., Chaiyadet S., Tripathi T., Laha T., Loukas A. (2018). Opisthorchis viverrini Proteome and Host-Parasite Interactions. Adv. Parasitol..

[128] Zheng Y. (2017). Proteomic analysis of *Taenia hydatigena* cyst fluid reveals unique internal microenvironment. Acta Trop..

[129] Dorey A., Cwiklinski K., Rooney J., De Marco Verissimo C., López Corrales J., Jewhurst H., Fazekas B., Calvani N., Hamon S., Gaughan S. (2021). Autonomous Non Antioxidant Roles for *Fasciola hepatica* Secreted Thioredoxin-1 and Peroxiredoxin-1. Front. Cell Infect. Microbiol..

[130] Al-Shehri S.S. (2021). Reactive oxygen and nitrogen species and innate immune response. Biochimie.

[131] Vinogradov A.D., Grivennikova V.G. (2016). Oxidation of NADH and ROS production by respiratory complex I. Biochim. Biophys. Acta.

[132] Moné Y., Ribou A.C., Cosseau C., Duval D., Théron A., Mitta G., Gourbal B. (2011). An example of molecular co-evolution: Reactive oxygen species (ROS) and ROS scavenger levels in *Schistosoma mansoni*/*Biomphalaria glabrata* interactions. Int. J. Parasitol..

[133] Berriman M., Haas B.J., LoVerde P.T., Wilson R.A., Dillon G.P., Cerqueira G.C., Mashiyama S.T., Al-Lazikani B., Andrade L.F., Ashton P.D. (2009). The genome of the blood fluke *Schistosoma mansoni*. Nature.

[134] Zhang H.C., Ma K.X., Yang Y.J., Shi C.Y., Chen G.W., Liu D.Z. (2018). Molecular cloning, characterization, expression and enzyme activity of catalase from planarian *Dugesia japonica* in response to environmental pollutants. Ecotoxicol. Environ. Saf..

[135] Hernández-Santoyo A., Landa A., González-Mondragón E., Pedraza-Escalona M., Parra-Unda R., Rodríguez-Romero A. (2011). Crystal structure of Cu/Zn superoxide dismutase from *Taenia solium* reveals metal-mediated self-assembly. FEBS J..

[136] Yang D., Fu Y., Wu X., Xie Y., Nie H., Chen L., Nong X., Gu X., Wang S., Peng X. (2012). Annotation of the transcriptome from Taenia pisiformis and its comparative analysis with three Taeniidae species. PLoS ONE.

[137] Mei H., LoVerde P.T. (1997). *Schistosoma mansoni*: The developmental regulation and immunolocalization of antioxidant enzymes. Exp. Parasitol..

[138] Toppo S., Vanin S., Bosello V., Tosatto S.C. (2008). Evolutionary and structural insights into the multifaceted glutathione peroxidase (Gpx) superfamily. Antioxid. Redox Signal..

[139] Changklungmoa N., Chaithirayanon K., Cheukamud W., Chaiwichien A., Osotprasit S., Samrit T., Sobhon P., Kueakhai P. (2018). Expression and characterization of glutathione peroxidase of the liver fluke. Parasitol. Res..

[140] Fan J., Wu H., Li K., Liu X., Tan Q., Cao W., Liang B., Ye B. (2020). Transcriptomic Features of *Echinococcus granulosus* Protoscolex during the Encystation Process. Korean J. Parasitol..

[141] Zelck U.E., Von Janowsky B. (2004). Antioxidant enzymes in intramolluscan *Schistosoma mansoni* and ROS-induced changes in expression. Parasitology.

[142] Cai G.B., Bae Y.A., Kim S.H., Sohn W.M., Lee Y.S., Jiang M.S., Kim T.S., Kong Y. (2008). Vitellocyte-specific expression of phospholipid hydroperoxide glutathione peroxidases in *Clonorchis sinensis*. Int. J. Parasitol..

[143] Low F.M., Hampton M.B., Winterbourn C.C. (2008). Peroxiredoxin 2 and peroxide metabolism in the erythrocyte. Antioxid. Redox Signal..

[144] Wang H., Li J., Zhang C., Guo B., Wei Q., Li L., Yang N., Peter McManus D., Gao X., Zhang W. (2018). *Echinococcus granulosus* sensu stricto: Silencing of thioredoxin peroxidase impairs the differentiation of protoscoleces into metacestodes. Parasite.

[145] Kumagai T., Osada Y., Kanazawa T. (2006). 2-Cys peroxiredoxins from Schistosoma japonicum: The expression profile and localization in the life cycle. Mol. Biochem. Parasitol..

[146] Threadgold L.T., Arme C., Read C.P. (1968). Ultrastructure localization of a peroxidase in the tapeworm, *Hymenolepis diminuta*. J. Parasitol..

[147] Circu M.L., Aw T.Y. (2011). Redox biology of the intestine. Free Radic. Res..

[148] Sun Q.A., Kirnarsky L., Sherman S., Gladyshev V.N. (2001). Selenoprotein oxidoreductase with specificity for thioredoxin and glutathione systems. Proc. Natl. Acad. Sci. USA.

[149] Su D., Novoselov S.V., Sun Q.A., Moustafa M.E., Zhou Y., Oko R., Hatfield D.L., Gladyshev V.N. (2005). Mammalian selenoprotein thioredoxin-glutathione reductase. Roles in disulfide bond formation and sperm maturation. J. Biol. Chem..

[150] Alger H.M., Williams D.L. (2002). The disulfide redox system of *Schistosoma mansoni* and the importance of a multifunctional enzyme, thioredoxin glutathione reductase. Mol. Biochem. Parasitol..

[151] Agorio A., Chalar C., Cardozo S., Salinas G. (2003). Alternative mRNAs arising from trans-splicing code for mitochondrial and cytosolic variants of *Echinococcus granulosus* thioredoxin Glutathione reductase. J. Biol. Chem..

[152] Rendón J.L., del Arenal I.P., Guevara-Flores A., Uribe A., Plancarte A., Mendoza-Hernández G. (2004). Purification, characterization and kinetic properties of the multifunctional thioredoxin-glutathione reductase from *Taenia crassiceps* metacestode (cysticerci). Mol. Biochem. Parasitol..

[153] Otero L., Bonilla M., Protasio A.V., Fernández C., Gladyshev V.N., Salinas G. (2010). Thioredoxin and glutathione systems differ in parasitic and free-living platyhelminths. BMC Genom..

[154] Martínez-González J.J., Guevara-Flores A., Alvarez G., Rendón-Gómez J.L., Del Arenal I.P. (2010). In vitro killing action of auranofin on *Taenia crassiceps* metacestode (cysticerci) and inactivation of thioredoxin-glutathione reductase (TGR). Parasitol. Res..

[155] Prast-Nielsen S., Huang H.H., Williams D.L. (2011). Thioredoxin glutathione reductase: Its role in redox biology and potential as a target for drugs against neglected diseases. Biochim. Biophys. Acta.

[156] Song L., Li J., Xie S., Qian C., Wang J., Zhang W., Yin X., Hua Z., Yu C. (2012). Thioredoxin glutathione reductase as a novel drug target: Evidence from Schistosoma japonicum. PLoS ONE.

[157] Eweas A.F., Allam G. (2018). Targeting thioredoxin glutathione reductase as a potential antischistosomal drug target. Mol. Biochem. Parasitol..

[158] Shukla R., Shukla H., Kalita P., Tripathi T. (2018). Structural insights into natural compounds as inhibitors of Fasciola gigantica thioredoxin glutathione reductase. J. Cell Biochem..

[159] Guevara-Flores A., Martínez-González J.J., Herrera-Juárez Á.M., Rendón J.L., González-Andrade M., Torres Durán P.V., Enríquez-Habib R.G., Del Arenal Mena I.P. (2019). Effect of curcuminoids and curcumin derivate products on thioredoxin-glutathione reductase from *Taenia crassiceps* cysticerci. Evidence suggesting a curcumin oxidation product as a suitable inhibitor. PLoS ONE.

[160] Lyu H., Petukhov P.A., Banta P.R., Jadhav A., Lea W.A., Cheng Q., Arnér E., Simeonov A., Thatcher G., Angelucci F. (2020). Characterization of Lead Compounds Targeting the Selenoprotein Thioredoxin Glutathione Reductase for Treatment of Schistosomiasis. ACS Infec. Dis..

[161] Faixová D., Hrčková G., Mačák Kubašková T., Mudroňová D. (2021). Antiparasitic Effects of Selected Isoflavones on Flatworms. Helminthologia.

[162] Cwiklinski K., Dalton J.P., Dufresne P.J., La Course J., Williams D.J., Hodgkinson J., Paterson S. (2015). The *Fasciola hepatica* genome: Gene duplication and polymorphism reveals adaptation to the host environment and the capacity for rapid evolution. Genome Biol..

[163] Bonilla M., Denicola A., Novoselov S.V., Turanov A.A., Protasio A., Izmendi D., Gladyshev V.N., Salinas G. (2008). Platyhelminth mitochondrial and cytosolic redox homeostasis is controlled by a single thioredoxin glutathione reductase and dependent on selenium and glutathione. J. Biol. Chem..

[164] Guevara-Flores A., Del Arenal I.P., Mendoza-Hernández G., Pardo J.P., Flores-Herrera O., Rendón J.L. (2010). Mitochondrial Thioredoxin-Glutathione Reductase from Larval *Taenia crassiceps* (Cysticerci). J. Parasitol. Res..

[165] Martínez-González J.J., Guevara-Flores A., Rendón J.L., Arenal I. (2015). Auranofin-induced oxidative stress causes redistribution of the glutathione pool in *Taenia crassiceps* cysticerci. Mol. Biochem. Parasitol..

[166] Kuntz A.N., Davioud-Charvet E., Sayed A.A., Califf L.L., Dessolin J., Arnér E.S., Williams D.L. (2007). Thioredoxin glutathione reductase from *Schistosoma mansoni*: An essential parasite enzyme and a key drug target. PLoS Med..

[167] Angelucci F., Miele A.E., Boumis G., Dimastrogiovanni D., Brunori M., Bellelli A. (2008). Glutathione reductase and thioredoxin reductase at the crossroad: The structure of *Schistosoma mansoni* thioredoxin glutathione reductase. Proteins.

[168] Hayes J.D., Flanagan J.U., Jowsey I.R. (2005). Glutathione transferases. Annu. Rev. Pharmacol. Toxicol..

[169] Wolkoff A.W. (1980). The glutathione S-transferases: Their role in the transport of organic anions from blood to bile. Int. Rev. Physiol..

[170] Nguyen H.A., Bae Y.A., Lee E.G., Kim S.H., Diaz-Camacho S.P., Nawa Y., Kang I., Kong Y. (2010). A novel sigma-like glutathione transferase of *Taenia solium* metacestode. Int. J. Parasitol..

[171] Pearson W.R. (2005). Phylogenies of glutathione transferase families. Methods Enzymol..

[172] Wu B., Dong D. (2012). Human cytosolic glutathione transferases: Structure, function, and drug discovery. Trends Pharmacol. Sci..

[173] Bae Y.A., Kim J.G., Kong Y. (2016). Phylogenetic characterization of *Clonorchis sinensis* proteins homologous to the sigma-class glutathione transferase and their differential expression profiles. Mol. Biochem. Parasitol..

[174] Iriarte A., Arbildi P., La-Rocca S., Musto H., Fernández V. (2012). Identification of novel glutathione transferases in *Echinococcus granulosus*. An evolutionary perspective. Acta Trop..

[175] Kim J.G., Ahn C.S., Kim S.H., Bae Y.A., Kwon N.Y., Kang I., Yang H.J., Sohn W.M., Kong Y. (2016). *Clonorchis sinensis* omega-class glutathione transferases play major roles in the protection of the reproductive system during maturation and the response to oxidative stress. Parasites Vectors.

[176] Ziniel P.D., Karumudi B., Barnard A.H., Fisher E.M., Thatcher G.R., Podust L.M., Williams D.L. (2015). The *Schistosoma mansoni* Cytochrome P450 (CYP3050A1) Is Essential for Worm Survival and Egg Development. PLoS Negl. Trop. Dis..

[177] Pakharukova M.Y., Vavilin V.A., Sripa B., Laha T., Brindley P.J., Mordvinov V.A. (2015). Functional Analysis of the Unique Cytochrome P450 of the Liver Fluke Opisthorchis felineus. PLoS Negl. Trop. Dis..

[178] Pal R., Rai J.P. (2010). Phytochelatins: Peptides involved in heavy metal detoxification. Appl. Biochem. Biotechnol..

[179] Rea P.A. (2012). Phytochelatin synthase: Of a protease a peptide polymerase made. Physiol. Plant..

[180] Ray D., Williams D.L. (2011). Characterization of the phytochelatin synthase of *Schistosoma mansoni*. PLoS Negl. Trop. Dis..

[181] Rigouin C., Vermeire J.J., Nylin E., Williams D.L. (2013). Characterization of the phytochelatin synthase from the human parasitic nematode Ancylostoma ceylanicum. Mol. Biochem. Parasitol..

[182] Williams D.L., Bonilla M., Gladyshev V.N., Salinas G. (2013). Thioredoxin glutathione reductase-dependent redox networks in platyhelminth parasites. Antioxid. Redox Signal..

[183] Bundy J.G., Kille P., Liebeke M., Spurgeon D.J. (2014). Metallothioneins may not be enough--the role of phytochelatins in invertebrate metal detoxification. Environ. Sci. Technol..

[184] Mannino M.H., Patel R.S., Eccardt A.M., Perez Magnelli R.A., Robinson C., Janowiak B.E., Warren D.E., Fisher J.S. (2019). Myoglobin as a versatile peroxidase: Implications for a more important role for vertebrate striated muscle in antioxidant defense. Comp. Biochem. Physiol. B Biochem. Mol. Biol..

[185] Ren M., He L., Huang Y., Mao Q., Li S., Qu H., Bian M., Liang P., Chen X., Ling J. (2014). Molecular characterization of *Clonorchis sinensis* secretory myoglobin: Delineating its role in anti-oxidative survival. Parasites Vectors.

[186] Penning T.M. (2015). The aldo-keto reductases (AKRs): Overview. Chem. Biol. Interact..

[187] Forrest G.L., González B. (2000). Carbonyl reductase. Chem. Biol. Interact..

[188] Escobedo G., Romano M.C., Morales-Montor J. (2009). Differential in vitro effects of insulin on *Taenia crassiceps* and *Taenia solium* cysticerci. J. Helminthol..

[189] Adalid-Peralta L., Rosas G., Arce-Sillas A., Bobes R.J., Cárdenas G., Hernández M., Trejo C., Meneses G., Hernández B., Estrada K. (2017). Effect of Transforming Growth Factor-β upon *Taenia solium* and *Taenia crassiceps* Cysticerci. Sci. Rep..

[190] Navarrete-Perea J., Moguel B., Mendoza-Hernández G., Fragoso G., Sciutto E., Bobes R.J., Laclette J.P. (2014). Identification and quantification of host proteins in the vesicular fluid of porcine *Taenia solium* cysticerci. Exp. Parasitol..

[191] Ahn C.S., Han X., Bae Y.A., Ma X., Kim J.T., Cai H., Yang H.J., Kang I., Wang H., Kong Y. (2015). Alteration of immunoproteome profile of *Echinococcus granulosus* hydatid fluid with progression of cystic echinococcosis. Parasites Vectors.

[192] Ahn C.S., Kim J.G., Han X., Kang I., Kong Y. (2017). Comparison of *Echinococcus multilocularis* and *Echinococcus granulosus* hydatid fluid proteome provides molecular strategies for specialized host-parasite interactions. Oncotarget.

[193] Flores-Bautista J., Navarrete-Perea J., Fragoso G., Flisser A., Soberón X., Laclette J.P. (2018). Fate of uptaken host proteins in *Taenia solium* and *Taenia crassiceps* cysticerci. Biosci. Rep..

[194] Ahn C.S., Kim J.G., Bae Y.A., Kim S.H., Shin J.H., Yang Y., Kang I., Kong Y. (2017). Fasciclin-calcareous corpuscle binary complex mediated protein-protein interactions in *Taenia solium* metacestode. Parasites Vectors.

[195] Loos J.A., Caparros P.A., Nicolao M.C., Denegri G.M., Cumino A.C. (2014). Identification and pharmacological induction of autophagy in the larval stages of *Echinococcus granulosus*: An active catabolic process in calcareous corpuscles. Int. J. Parasitol..

[196] Cwiklinski K., Robinson M.W., Donnelly S., Dalton J.P. (2021). Complementary transcriptomic and proteomic analyses reveal the cellular and molecular processes that drive growth and development of *Fasciola hepatica* in the host liver. BMC Genom..

[197] Becerro-Recio D., González-Miguel J., Ucero A., Sotillo J., Martínez-Moreno Á., Pérez-Arévalo J., Cwiklinski K., Dalton J.P., Siles-Lucas M. (2021). Recognition Pattern of the *Fasciola hepatica* Excretome/Secretome during the Course of an Experimental Infection in Sheep by 2D Immunoproteomics. Pathogens.

[198] Virginio V.G., Monteiro K.M., Drumond F., de Carvalho M.O., Vargas D.M., Zaha A., Ferreira H.B. (2012). Excretory/secretory products from in vitro-cultured *Echinococcus granulosus* protoscoleces. Mol. Biochem. Parasitol..

[199] Wang H., Zhang C.S., Fang B.B., Li Z.D., Li L., Bi X.J., Li W.D., Zhang N., Lin R.Y., Wen H. (2019). Thioredoxin peroxidase secreted by *Echinococcus granulosus* (sensu stricto) promotes the alternative activation of macrophages via PI3K/AKT/mTOR pathway. Parasites Vectors.

[200] García-Montoya G.M., Mesa-Arango J.A., Isaza-Agudelo J.P., Agudelo-Lopez S.P., Cabarcas F., Barrera L.F., Alzate J.F. (2016). Transcriptome profiling of the cysticercus stage of the laboratory model *Taenia crassiceps*, strain ORF. Acta Trop..

[201] Greenberg R.M. (2013). ABC multidrug transporters in schistosomes and other parasitic flatworms. Parasitol. Int..

